# Not When But Whether: Modality and Future Time Reference in English and Dutch

**DOI:** 10.1111/cogs.13224

**Published:** 2023-01-19

**Authors:** Cole Robertson, Seán G. Roberts

**Affiliations:** ^1^ Centre for Language Studies Radboud University; ^2^ School of English, Communication and Philosophy Cardiff University

**Keywords:** Future time reference, Psychological discounting, Modality, Future tense, Linguistic relativity, Linguistic savings hypothesis

## Abstract

Previous research on linguistic relativity and economic decisions hypothesized that speakers of languages with obligatory tense marking of future time reference (FTR) should value future rewards less than speakers of languages which permit present tense FTR. This was hypothesized on the basis of obligatory linguistic marking (e.g., *will*) causing speakers to construe future events as more temporally distal and thereby to exhibit increased “temporal discounting”: the subjective devaluation of outcomes as the delay until they will occur increases. However, several aspects of this hypothesis are incomplete. First, it overlooks the role of “modal” FTR structures which encode notions about the likelihood of future outcomes (e.g., *might*). This may influence “probability discounting”: the subjective devaluation of outcomes as the probability of their occurrence decreases. Second, the extent to which linguistic structures are subjectively related to temporal or probability discounting differences is currently unknown. To address these, we elicited FTR language and subjective ratings of temporal distance and probability from speakers of English, which exhibits strongly grammaticized FTR, and Dutch, which does not. Several findings went against the predictions of the previous hypothesis: Framing an FTR statement in the present (“Ellie arrives later on”) versus the future tense (“…will arrive…”) did not affect ratings of temporal distance; English speakers rated future statements as relatively more temporally proximal than Dutch speakers; and English and Dutch speakers rated future tenses as encoding high certainty, which suggests that obligatory future tense marking might result in less discounting. Additionally, compared with Dutch speakers, English speakers used more low‐certainty terms in general (e.g., *may*) and as a function of various experimental factors. We conclude that the prior cross‐linguistic observations of the link between FTR and psychological discounting may be caused by the connection between low‐certainty modal structures and probability discounting, rather than future tense and temporality.

## 1 Introduction

Do differences between languages change the way people think, feel, and act? The linguistic relativity hypothesis suggests that they do (Whorf, 1956; also see Gumperz & Levinson, 1996; Leavitt, 2011; Lucy, 1992). The idea is that languages force speakers to notice different things in order to communicate and that the resultant differences in online attentional demands can grow through lifelong language use into entrenched offline cognitive differences (Wolff & Holmes, 2011). For instance, when choosing between the English demonstratives *this* and *that*, speakers need only pay attention to whether the referred‐to object is located near or far from themselves. Spanish breaks this space into three degrees of distance: *este* ‘this,’ *ese* ‘that,’ and *aquél* ‘that’ (distant, i.e., ‘yon’ [archaic]). Malagasy breaks it into seven (Evans, Bergqvist, & San Roque, 2018). Might speakers of Spanish or Malagasy be faster or more precise at estimating distance from ego? A growing body of research attests to affects like this (see Casasanto, 2016; Everett, 2013; Lupyan, Rahman, Boroditsky, & Clark, 2020; Majid, 2018; Wolff & Holmes, 2011).

A typical way linguistic relativity research progresses is by identifying cross‐linguistic differences and then investigating whether they give rise to corollary cognitive effects (Lucy, 1997, 2016). In this vein, economists have been exploring whether cross‐linguistic differences in the grammatical rules that apply when forming linguistic utterances about future events (future time reference, or FTR)^1^ affect speakers' subjective estimation of the value of delayed outcomes (for review, see, Mavisakalyan & Weber, 2018). This is referred to as “temporal discounting.” However, prior research of this kind has been criticized for its superficial treatment of FTR (Dahl, 2013; McWhorter, 2014; Pereltsvaig, 2011; Pullum, 2012; Sedivy, 2012). In this paper, we aim to develop a more comprehensive understanding of the relation between various strategies for talking about the future and the cognitive biases behind psychological discounting in order to help develop the linguistic savings hypothesis.

### 1.1 The linguistic savings hypothesis explained

The hypothesis that FTR grammaticization affects temporal discounting is referred to as the “linguistic savings hypothesis” (K. Chen, 2013). It is based on two observations. The first involves cross‐linguistic differences in whether a future tense *must* be used for FTR. For instance, In English and Dutch, future tenses are formed using paraphrastic auxiliary modal verbs (English *will, shall*; Dutch *zullen* ‘will’), or *de andative*—go‐based—constructions (English *be going to*; Dutch *gaan* ‘be going to’). However, English obliges speakers to use future tenses when referring to the future, while Dutch does not:

(1)
  a. English
*It will rain tomorrow*.
  b. Dutch *Morgen* *regent* *het*.
    tomorrow rain:PRS it
    ‘It will rain tomorrow.’

When English speakers make predictions about the future, it is necessary to use *will*, *be going to*, or *shall* (Dahl, 2000b), for example, (1a). No such restrictions exist in Dutch, where present tense constructions like (1b) are acceptable (Behydt, 2005). Based on typological research by Dahl (1985, 2000b) and his colleagues, K. Chen (2013) created a dichotomous typological variable which classes N=129 languages into two categories. Languages like Dutch with non‐obligatory future tenses are classed as “weak‐FTR.” Languages like English with obligatory future tenses are classed as “strong‐FTR.”^2^ Specifically, weak‐FTR languages are those languages which do not oblige the future tense in prediction‐based contexts. Strong‐FTR languages are those languages which are not weak‐FTR (K. Chen, 2013). In this context, prediction‐based FTR contexts are contrasted with schedule‐based and intention‐based ones (Dahl, 2000b). This is relevant because it is quite common for languages to permit the present tense when referring to schedules (Dahl, 2000b). English is a good example: *The Bears play at 7 pm* (schedule) is perfectly acceptable, while *The Bears win at 7 pm* (prediction) is not (Behydt, 2005). As such, when we refer to languages “obliging” the future tense, we refer only to prediction‐based FTR. We refer to K. Chen's (2013) dichotomous weak/strong distinction as “FTR status.”

The second observation is that people tend to temporally discount delayed future outcomes as the time until they will occur grows longer (Green, Myerson, & Vanderveldt, 2014; Rachlin, Raineri, & Cross, 1991). For example, most people would prefer $100 immediately, rather than $100 after a year‐long delay. Temporal discounting rates are variable. Offer $200 in a year, and some people will prefer the immediate $100, while others will wait for the $200. Such preferences depend on individual differences in temporal discounting rates (Green & Myerson, 2004). Less discounting means higher estimations of delayed value. More discounting means lower estimations. Therefore, people with high temporal discounting rates tend to make “present‐oriented” decisions not to wait. People with low rates tend to make “future‐oriented” decisions to wait. It is common to refer to such differences in terms of “time preferences.”

**Fig. 1:**
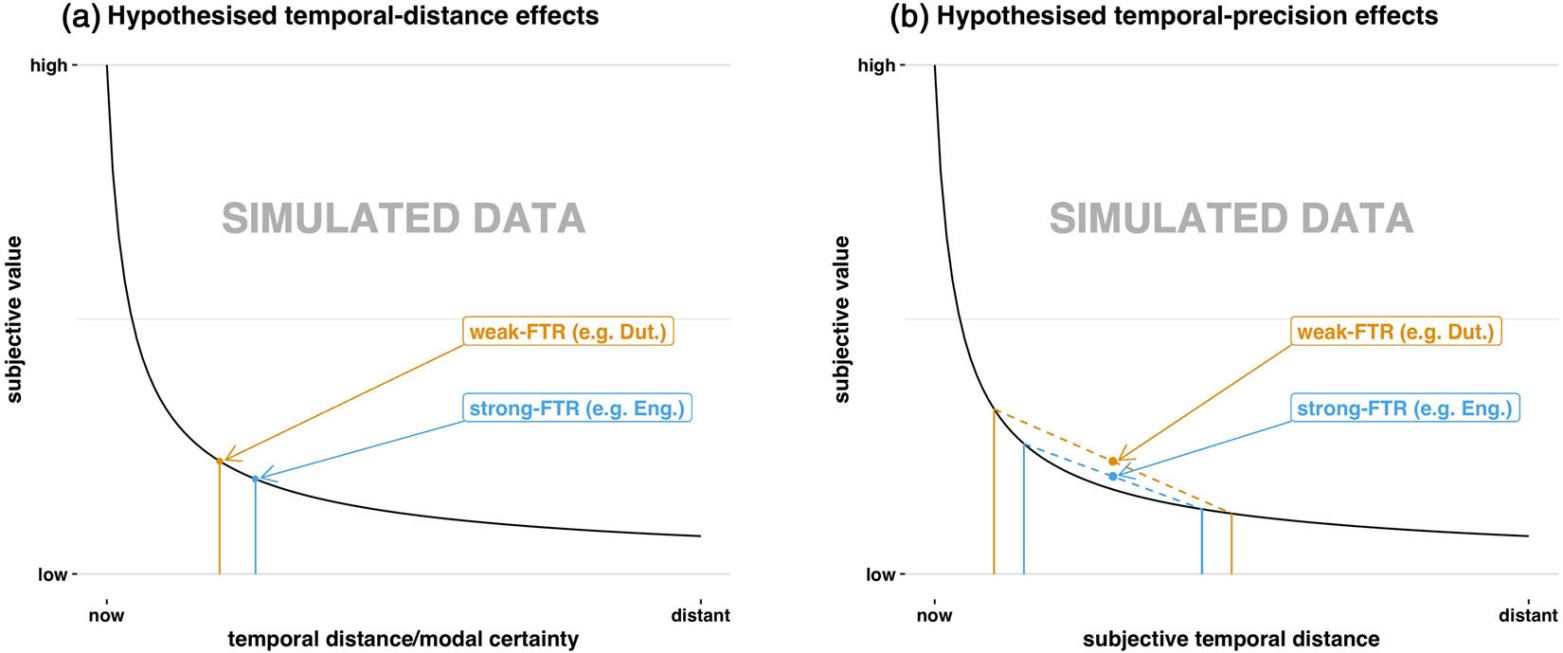
Mechanisms by which FTR grammaticization is hypothesized to affect temporal beliefs and therefore discounting. K. Chen (2013) hypothesized that speakers of weak‐FTR languages would construe future events as more temporally proximal (a) or less temporally precise (b). In (a), distal representations lead to decreased relative subjective value in strong‐FTR speakers; in (b), more precise temporal representations lead to relatively lower average subjective value in strong‐FTR speakers. We have presented the mechanisms in simplified terms. The distance mechanism is presented as a point estimate (a), and the precision mechanism is presented as the mean of a two‐item uniform distribution (b). In K. K. Chen's (2013) account, temporal beliefs are represented as normal distributions and subjective values are integrals. The discounting function plotted is a hyperboloid function, $V=A/{\left(1+kD\right)}^{s}$, from Green and Myerson (2004), where *V* is subjective value, *A* is the objective amount, *D* is the delay, *b* is a parameter that governs discounting rate, and *s* is a non‐linear scaling factor typically less than 1. This function has been found to accurately describe empirical discounting rates in humans (Du, Green, & Myerson, 2002; Green & Myerson, 2004; Green, Myerson, & Vanderveldt, 2014; Vanderveldt, Green, & Myerson, 2015). Plotted values for *s* and *k* are approximately average human discounting rates for the given delay *D* (0–100 months) and value *V* ($200 in this case), that is, s=0.7 and k=0.4 (Green & Myerson, 2004, from).

The linguistic savings hypothesis predicts that speakers of weak‐FTR languages like Dutch will temporally discount less than speakers of strong‐FTR languages like English. This implies that speakers of weak‐FTR languages will tend to make more future‐oriented decisions because they construe future rewards as relatively more valuable. K. Chen (2013) hypothesized that there were two distinct mechanisms which might explain this. Both involve differences in underlying beliefs about the temporal “location” of future events (K. Chen, 2013). The first is that the present tense conveys a sense of temporal immediacy. Since speakers of weak‐FTR languages often use the present tense for FTR, K. Chen (2013) hypothesized this would cause them to perceive future events as relatively temporally proximal (Fig. 1a). Second, he hypothesized weak‐FTR languages might not mandate that speakers think as precisely about the temporal location of future events (Fig. 1b). The idea is that strong‐FTR languages divide the “arrow of time” into three segments (past vs. present vs. future). Weak‐FTR languages divide it into two (past vs. present + future). K. Chen (2013) hypothesized that this finer segmentation in strong‐FTR languages causes more precise temporal representations of future events (K. Chen, 2013). If beliefs are affected in either of these ways, it would lead to relatively less discounting in weak‐FTR speakers (see Fig. 1). This would cause speakers of weak‐FTR languages to be more future oriented (K. Chen, 2013).

Such differences in future orientation reliably predict real‐world “intertemporal decisions,” in which individuals balance present versus future costs and rewards. For instance, time preferences have been found to predict real spending (Bickel et al., 2010) and financial outcomes such as income levels and financial mismanagement (Hamilton & Potenza, 2012; Xiao & Porto, 2019). Time preferences also predict substance abuse tendencies, which often incur long‐term costs (professional, social) but confer short‐term benefits (hedonistic pleasure). This includes alcohol abuse (Vuchinich & Simpson, 1998), opioid dependency (Garami & Moustafa, 2019), and substance abuse in general (Kirby, Petry, & Bickel, 1999; Mejía‐Cruz, Green, Myerson, Morales‐Chainé, & Nieto, 2016). Health behaviors are often impacted as well, because many heath‐critical decisions involve trade‐offs between immediate (dis)comfort and future (ill)health. For instance, time preferences predicted the odds of smoking cigarettes (Bickel, Odum, & Madden, 1999) and the likelihood of exercising in older individuals (Tate, Tsai, Landes, Rettiganti, & Lefler, 2015). Therefore, compared with speakers of strong‐FTR languages like English, speakers of weak‐FTR languages like Dutch are predicted to save more for the future, exercise more, and make healthier lifestyle choices.

K. Chen (2013), tested these predictions by using FTR status in regression analyses to predict a range of behaviors. He found that speakers of weak‐FTR languages were more likely to have saved each year, retired with more assets, were less likely to have smoked, and were more likely to practice safe sex. He also found they were healthier, as indexed by obesity, peak blood flow, grip strength, and physical exercise levels. Since then, numerous studies have extended this basic approach. For example, speakers of weak‐FTR languages engaged less in present‐oriented accounting practices (Fasan, Gotti, Kang, & Liu, 2016; J. Kim, Kim, & Zhou, 2017), had better educational outcomes (Figlio et al., 2016), made healthier lifestyle choices (Guin, 2017), had greater support for future‐orientated environmental policies (Mavisakalyan, Tarverdi, & Weber, 2018; Pérez & Tavits, 2017), and had better macroeconomic performance (Hübner & Vannoorenberghe, 2015a, 2015b). A number of other studies attest to the conclusion that FTR status is a reliable predictor of intertemporal behavior (S. Chen, Cronqvist, Ni, & Zhang, 2017; Chi, Su, Tang, & Xu, 2018; Galor et al., 2016; Liang et al., 2018; Lien & Zhang, 2020; Sutter, Angerer, Glätzle‐rützler, & Lergetporer, 2015; Thoma & Tytus, 2018). Although there are various statistical concerns with the robustness of these associations (Gotti, Roberts, Fasan, & Robertson, 2021; Roberts, Winters, & Chen, 2015), practically all studies make simplified assumptions about FTR typology. We now turn to some criticisms of these assumptions.

### 1.2 Critical perspectives on the linguistic savings hypothesis

In this section, we outline three issues with the theory and evidence for the linguistic savings hypothesis. These are (a) probability may be a confounding factor in observed effects of FTR status, (b) modal FTR expressions are disregarded despite being an import way of talking about the future, and (c) temporal accounts of the future tense disregard modal semantics of future tenses themselves.

#### 1.2.1 Probability may confound observed findings

A serious issue is that (as far as we know), no work has directly tested the temporal mechanisms proposed by K. Chen (2013). Regression analyses which use FTR status to predict real‐world intertemporal behavior cannot identify whether temporal or probability discounting is driving outcomes. Probability discounting is analogous to temporal discounting. It refers to the subjective devaluation of outcomes as their odds of occurring reduce (Green et al., 2014; Rachlin et al., 1991). For example, most people would prefer $100, over a 50% chance of receiving $100. However, offer a 50% chance of $200, and some will choose to gamble while others will choose the guaranteed $100. Differences like this are referred to in terms of “risk preferences.” Recently, there has been an increasing interest in investigating outcomes which are both delayed and risky, for example, $100 or a 50% chance of $200 in a year (Luckman, Donkin, & Newell, 2018; Vanderveldt et al., 2015; Vanderveldt, Green, & Rachlin, 2017). These are referred to as “risky intertemporal decisions.”

Many (if not all) of the behaviors found to be predicted by FTR status involve risky intertemporal decision‐making. Even nominally risk‐free outcomes usually involve some degree of uncertainty. For instance, the pursuit of educational goals is fraught with uncertainty about their relative rate of return (Figlio et al., 2016). The discounting of future suffering in the context of support for euthanasia is permeated with uncertainty about the relative extent of future suffering (Lien & Zhang, 2020). And accountants undertaking earnings management must weight the probability of being caught (Fasan et al., 2016; Kim et al., 2017). Even the main finding in K. Chen (2013) involves predicting whether survey respondents had saved in the past year, which could have involved investment in risky assets such as stocks and shares (World Values Survey Association, 2014).

Critically, probability and delay have been found to interactively predict subjective estimations of future value (Vanderveldt et al., 2015, 2017). Models which combine these factors fitted empirical results better than models which isolate them (Luckman et al., 2018). The probability of a reward had a greater impact on temporal discounting rates than delay has on probability discounting (Vanderveldt et al., 2015). These results support the conclusion that probability and delay interact to inform intertemporal decision‐making. This is a critical issue for the linguistic savings hypothesis. FTR status has been found to predict a range of behaviors. However, the nature of the outcomes makes it unclear why this is the case. Is probability or temporal discounting driving results?

#### 1.2.2 FTR‐status and modal future time reference

K. Chen (2013) uses obligatory tense marking of prediction‐based FTR as a proxy for FTR grammaticization . This may be reasonable (Dahl, 2000b), but *what* is it a proxy for? The expression of future time is very complex and often involves the expression of modal notions of ability, desire, (un)certainty, probability, volition, intention, and obligation (Bybee & Dahl, 1989; Bybee et al., 1994; Fries, 1956; Palmer, 2001, see). Modality involves quantifying what is likely—unlikely, or possible—necessary, relative to various modal “bases” (Kratzer, 1977; Palmer, 2001). For instance, *deontic* modality involves expressing what is desirable or necessary relative to social norms, taboos, and institutions (Palmer, 2001), for example, *One should always get up early*. In *epistemic* modality, speakers express what is likely relative to what they know or believe (Palmer, 2001), for example, *I really think he's got a chance!*

The grammaticization of FTR can involve multidimensional obligatorization processes, which involve many of these domains simultaneously becoming more grammaticized (Hopper, 1996). Epistemic modality is of critical relevance to questions of psychological discounting. Risky intertemporal preferences are impacted by the perceived likelihood of a future outcome. The obligation to use low‐certainty modal FTR constructions might cause strong‐FTR speakers to construe future events as more risky. In suggesting this, we are sympathetic to accounts which treat modal expressions as scalar operators which map transparently onto notions of probability. Rather than traditional accounts which invoke Boolean quantification (Kratzer, 2012), modal semantics are seen as encoding the likelihood of events on a one‐dimensional scale between high (p=1) and low (p=.5) certainty.^3^ Evidence suggests scalar accounts capture modal semantics better than notions of Boolean quantification since the latter yields incorrect predictions in some linguistic contexts (Lassiter, 2015). With this in mind, it is uncontroversial that modal constructions encode weakened certainty relative to the future tense (Enç, 1996; Huddleston Pullum, 2002; & Palmer, 2001). If such operators map onto scalar notions of probability, the obligation to use “low‐probability” modal constructions could cause strong‐FTR speakers to construe risky future outcomes as having a lower probability of occurring and therefore as less valuable.

This is problematic because FTR status affects the extent to which languages oblige the encoding of low‐certainty epistemic modality. For instance, *will* is not actually obligatory for prediction‐based FTR. Rather, English obliges speakers to use *will* or another modal verb:

(2)
  a. *The Bears* will *win (tonight)*.
  b. [If they get their defence together…]^4^ *…the Bears* would *win (tonight)*.
  c. *The Bears can win (tonight)*.
  d. *The Bears could win (tonight)*.
  e. *The Bears may win (tonight)*.
  f. *The Bears might win (tonight)*.
  g. *The Bears shall win (tonight)*.
  h. *The Bears should win (tonight)*.

Any of examples (2a–h) are perfectly acceptable. These modal verbs all encode futurity but express differing speaker commitment to the probability of the event occurring (Karawani & Waldon, 2017). If the English case generalizes, the salient difference between strong‐ and weak‐FTR languages might be that strong‐FTR languages oblige speakers to use a modal verb to encode *whether* they think an event will occur. If this results in more frequent net use, low‐certainty linguistic structures, such linguistic spotlighting, might cause increased probability discounting in strong‐FTR speakers.

Critically, it is unclear whether the grammatical distinction noted above actually results in more frequent use of low‐certainty language in English FTR as compared to Dutch. In Dutch, *kunnen* ‘may’ is the only modal verb for which epistemic use is possible and encodes possibility (Nuyts, 2000). *Kunnen* is not obligatory for prediction‐based FTR, whereas the English modals are. It seems plausible that this results in higher encoding of low‐certainty modality in English. However, Dutch speakers might be making up for language‐level grammatical constraints by expressing low certainty in other ways. For instance, in English and Dutch, epistemic modality can be expressed using modal modifiers, for example, English *possibly, probably, certainly*; Dutch *mogelijk (erwijze)* ‘possibly,’ *waarschijnlijk* ‘probably,’ *zeker* ‘certainly.’ Mental state predicates might also facilitate the expression of complex modal notions about the future. These are psychological verbs which allow speakers to express modal notions by talking about their thoughts and beliefs (Nuyts, 2000). In English and Dutch, the mental state predicate prototypically used to express epistemic modality is *think* (Dutch *denken* ‘think’), while *believe* (Dutch *geloven* ‘believe’) is also fairly common (Nuyts, 2000, p. 110), while *know* (Dutch *weten* ‘know’) has a minor role (Nuyts, 2000, p. 130). There may also be modal FTR differences which cut across the FTR status dichotomy. For instance, Dutch has a system of modal particles which can attenuate other modal structures, for example, *wel eens* ‘well be’ (approximate) (Nuyts, 2000). The flavor of this can be seen in *well* in English. For instance, *That could well be the train arriving* communicates strengthened modality compared with *That could be the train arriving*. However, English lacks this word class. English and Dutch both exhibit sophisticated systems for expressing modal notions (see Nuyts, 2000). However, the relevant question to linguistic relativity is not what *may* be said but what *must* be said (Jakobson, 1971). The English modal system is obligatory. If this causes English speakers to use more low‐certainty modals in FTR, this could impact risky‐intertemporal preferences.

#### 1.2.3 Do future tenses encode time or modality?

A second issue is that future tense markers tend to be characterized by a division of labor between temporal and modal semantics (Dahl, 2000b). Tenses are usually thought of as deictic expressions, which relate the time of a referenced event to the time of speech (Lyons, 1968; Mezhevich, 2008). In a typical ternary account of tense, Klein (1995) proposes that tense clarifies the temporal order between the utterance time and the reference time, so for example, the present tense indicates reference time and utterance time are the same, past tense indicates reference time precedes utterance time, and the future tense indicates reference time follows utterance time. For instance, in English:

(3)
  a. **Past**: *It rained*.
  b. **Present**: *It is raining*.^5^
  c. **Future**: *It will/shall/is going to rain*.

What is being expressed in example 3a–c is when, relative to the time of utterance, the event in question takes place. Other theoretical treatments of tense eschew ternary models inspired by properties ascribed to time by contemporary physics (Broekhuis & Verkuyl, 2014). For instance, Te Winkel (1866), and later Verkuyl (2008), combines elements of tense and aspect in positing that there are eight Dutch tense forms based on three binary oppositions: (1) present versus past, (2) synchronous versus posterior, and (3) imperfect versus perfect. In the present–synchronous category, an imperfective statement would be *Elsa loopt* ‘Elsa walks’ (i.e., the simple present), while a perfective statement would be *Elsa heeft gelopen* ‘Elsa has walked’ (the present perfect); whereas in the present–posterior category, an imperfective statement would be *Elsa zal lopen* ‘Elsa will walk’ (simple future), and a perfective would be *Elsa zal hebben gelopen* ‘Elsa will have walked’ (future perfect) (examples from Broekhuis & Verkuyl, 2014). Thus, past/present distinguishes between what most would consider past and present tense, synchronous/posterior distinguishes between past + present on the one hand and future on the other, and perfective/imperfective distinguishes between the English simple and prefect aspect (which express deictic time relations relative to the time of reference rather than the time of utterance). These are two accounts of tense. The salient point is that tenses are semantically defined as those linguistic structures which encode notions about when in time events occurs relative to the time of speech (Lyons, 1968).

However, it is often difficult to account for future tense semantics entirely in the framework of deictic time relations. This is because future tenses tend to comprise a mixture of modal, temporal, and aspectual notions (Dahl, 2000b). To understand this discussion, it is necessary to understand what we refer to as “FTR mode.” FTR mode is a set of notions which are essential to understanding FTR. They delineate the contexts in which it is possible to refer to future events. As we have mentioned, these are (a) intentions, (b) predictions, and (c) schedules. We follow Dahl's (2000b) useful schema by defining these categories as follows. Intentions are statements about our own or other people's intentions for the future, for example, *I shall see what's behind that door*. Speakers can usually be fairly certain about their own intentions, because they have access to the internal contents of his own minds. Schedules are high‐certainty statements about well‐known scheduled events, for example, *the game is at 6 pm*. Predictions are statements about less well‐known events about which the speaker cannot be sure. For instance, *that coin will land on heads* is a prediction.

##### The modal semantics of the English *will*:

The case that *will* encodes modal weakening usually involves pointing out that it becomes increasingly obligatory as the implied certainty decreases from schedules, to intentions, to predictions:

(4)
  a. *Sun rise is/?will be at 6am*.
  b. *I set out/am setting out/will set out for the coast soon*.
  c. *The bomb ?explodes/?is exploding/will explode soon*. (Bouma, 1975)

These sentences are syntactically similar, and all refer to the future. However, *will* becomes obligatory as the FTR mode grows increasingly uncertain. In example (4a), *will* sounds out of place. It manages to convey an overly formal register, that is, as a *maître d'* might announce *Dinner will be served at 7*. While it is grammatical, it does not seem standard. In example (4b), *will* does not serve strictly as a marker of future time. Rather, the meaning changes as a matter of stress. In I *will set out for the coast…*, the speaker will go (as opposed to someone else). In *I will set out for the coast…*, the speaker in fact going (as opposed to not going at all). Apart from the use of *will* to express such notions, the present tense is likely more common. On the other hand, in example (4c), neither the present or the present progressive is grammatical. On the basis of acceptability judgments like these, it is usually suggested that *will* marks *prediction* rather than FTR (Enç, 1996; Dahl, 2000b; Huddleston, 1995; Fries, 1956; Klecha, 2014).

Such a conclusion is supported by the fact that it is perfectly acceptable to use *will* to mark a prediction in present time contexts. For instance, on hearing a knock at the door, it is grammatical to say either of:

(5)
  a. *That will be the postman*.
  b. *That is the postman*.

In example (5a), *will* marks a present time prediction. This suggests that the semantics of *will* are not strictly temporal. Rather, *will* tends to mark predictions regardless of the time frame (Enç, 1996; Giannakidou & Mari, 2018; Huddleston, 1995; Huddleston & Pullum, 2002; Klecha, 2014, inter alia:). Some commentators have pointed out that *will* may also operate as a marker of modal necessity, similarly to *must* (Giannakidou, 2017; Giannakidou & Mari, 2018). For instance, in example (5a), *will* expresses something similar to *that must be the postman*. A relevant point here is that such statements actually also express modal weakening relative to statements of fact (Giannakidou & Mari, 2018). In other words, *that must be the postman* implies that the speaker is inferring this, perhaps on the basis of relevant knowledge. If they knew it were the postman, they would just use example (5b).

For these and other reasons, most scholars agree that a purely temporal interpretation of *will* is inadequate, though the precise modal semantics of *will* are debated (Broekhuis & Verkuyl, 2014; Cariani & Santorio, 2018; Dahl, 2000b; Enç, 1996; Fries, 1956; Huddleston, 1995; Huddleston & Pullum, 2002; Klecha, 2014; Sarkar, 1998; Salkie, 2010). For instance, obliging the use of *will* for predictions may spotlight the uncertainty associated with this FTR mode. The meaning of *will* may be associated with its use, that is, it may “mean” epistemic weakening. On the other hand, there do not appear to be any convincing demonstrations that the modal weakening of *will* in example (5a) “carries over” when *will* is used to mark future predictions. It seems unclear that it would, given it is not possible to use the present tense for prediction‐based FTR in English. In fact, as we have pointed out, it is not actually obligatory to use *will* in example (5a). English speakers are rather obliged to use one of the English modals. A paradigmatic analysis of the options available indicates that *will* therefore encodes high certainty: It is among the highest certainty options available. This echoes suggestions that it is a marker of epistemic necessity (Giannakidou & Mari, 2018; Klecha, 2014).

##### The modal semantics of the Dutch *zullen*:

Similar debates are had about the theoretical status of the Dutch future, *zullen* ‘will.’ Is it a modal or a tense? Broekhuis and Verkuyl (2014) make the case that its semantics are only modal. The authors point out that the Dutch present tense can be used to refer to a time span encompassing both before (using the present perfect) and after the time of speech. On this basis, it is concluded that the contribution of *zullen* must be *purely* modal (Fehringer, 2018; Giannakidou, 2014, 2017). They give the following examples. The uncontroversial modal auxiliaries of possibility, *kunnen* ‘may,’ and necessity, *moeten* ‘must,’ are contrasted with *zullen* ‘will’:

(6) Dutch
  a. *Dat* *huis* *op* *de* *hoek* *moet* *instorten*.
    that house on the corner must collapse:PRS
    ‘That house on the corner must be collapsing.’
  b. *Dat* *huis* *op* *de* *hoek* *kan* *instorten*.
    that house on the corner may collapse:PRS
    ‘That house on the corner may be collapsing.’
  c. *Dat* *huis* *op* *de* *hoek* *zal* *instorten*.
    that house on the corner will collapse:PRS
    ‘That house on the corner will be collapsing.’

According to Broekhuis and Verkuyl (2014), examples (6a–c) are all compatible with a future reading. However, given concurrent evidence of a collapse actually occurring (i.e., rumbling, visible instability), they can also refer to a present time event (Broekhuis & Verkuyl, 2014). If both present and future time interpretations are possible for *zullen*, they suggest that its primary contribution cannot be temporal and must be *purely* modal.

This is probably an extreme position, but the more modest assertion that *zullen* encodes modal semantics appears uncontroversial. For instance, the *Algemene Nederlandse Spraakkunst* , which is a standard reference for Dutch speakers (Fehringer, 2018), indicates that *zullen* tends to encode low certainty, while greater certainty is expressed by *gaan* ‘be going to,’ though these differences may be limited to interrogative contexts (Geerts, Haeseryn, Romijn, de Rooij, & van den Toorn, 1997). Like the English *shall*, *zullen* grammaticized from a Germanic word meaning “to owe” (Dahl, 2000b, p. 319), and it historically retained a deontic flavor, expressing obligations and necessities (Fehringer, 2018), as well as epistemic supposition (Fehringer, 2018), and simple FTR (Behydt, 2005). Fehringer (2018) points out that, both synchronically and diachronically, it is difficult to disentangle *zullen*'s modal and temporal semantics, leading scholars to question whether a clear partition is even possible. As with English *be going to* future constructions, *gaan* emerged much later as a future marker and retains elements of its earlier “movement towards a goal” meaning. This may lend itself to the expression of intentions (Fehringer, 2018). At the same time, there may be differences in temporal semantics between *gaan* and *zullen*: Some scholars suggest the former may encode near, and the latter distal, future time (Behydt, 2005; Ten Cate, 1991) (the same observation has been made of English *be going to* versus *will*; Behydt, 2005; Royster & Steadman, 1923). There are also regional differences, for instance, *gaan* is more common and may be more grammaticized in West‐Flemish Dutch as compared to (Northern) Dutch (Behydt, 2005; Fehringer, 2018). Like *will*, modern *zullen* seems characterized by an admixture between modal and temporal semantics (Kirsner, 1969; also see: Janssen, 1989; Fehringer, 2018; Olmen, Mortelmans, & Auwera, 2009; Sluijs, 2011)—a statement that applies to many future “tenses.”

##### Comparing future tense semantics in English and Dutch:

As with *will*, the exact nature of the semantic contribution of *zullen* is difficult to pin down. Broekhuis and Verkuyl (2014) suggest *zullen* constitutes marking of an expected or “projected” future. A paradigmatic analysis is useful. In Dutch, it is possible to use the present tense for prediction‐based FTR (Behydt, 2005; Dahl, 2000b). The Dutch future‐reference present tense may encode complete certainty (Behydt, 2005). This suggests that *zullen* encodes modal weakening relative to present tense FTR. On the other hand, relative to *kunnen* ‘may,’ *zullen* appears to encode higher certainty. In contrast, the English future is the highest certainty option available for prediction‐based FTR. This means paradigmatic analyses of the *will* and *zullen* lead to different conclusions contingent on FTR status. The English future tense is the highest certainty construction possible for future predictions. On the other hand *zullen* and *gaan* may be paradigmatically contrasted present tense FTR. Relative to such unmarked statements of fact, any modalization is weaker. The paradigmatic oppositions of future tenses may therefore differ as a function of the cross‐linguistic differences indexed by FTR status. On the other hand, if *will* and *zullen* are markers of futurity, serving to move reference time posterior to utterance time, then, by this account, their semantics are both high certainty .

### 1.3 Implications for linguistic relativity

Relativity accounts of how FTR grammaticization impacts (risky) intertemporal decisions need to confront these evident complexities. Critically, K. Chen's (2013) arguments ignore the division of labor between temporal and modal semantics which often characterizes future “tenses.” We have outlined plausible arguments that *will* and *zullen* encode either modal strengthening or weakening. Which of these accounts is closer to reality has important implications. If modal weakening is encoded, obligatory future tenses should cause speakers to perceive the future as less certain. They would therefore discount more. If modal strengthening is encoded, future outcomes might be construed as more certain. Speakers would therefore discount less. Additionally, cross‐linguistic differences in future “tense” semantics undermine K. Chen's (2013) argument that obligatory use of the future tense should impact speakers of different languages in the same way.

In summary, both probability and delay affect subjective estimations of value (Białaszek, Ostaszewski, Green, & Myerson, 2019; Green, Myerson, & Ostaszewski, 1999; Green and Myerson, 2004; Rachlin et al., 1991). There is a robust typological tendency for future tenses to encode modal notions (Broekhuis & Verkuyl, 2014; Bybee et al., 1994; Enç, 1996; Fries, 1956; Giannakidou, 2014; Giannakidou & Mari, 2018; Huddleston, 1995; Nuyts & Vonk, 1999; Sarkar, 1998). Additionally, modal verbs themselves are cross‐linguistically common FTR structures which allow speakers to encode degrees of epistemic commitment to future events. Strong FTR languages may oblige the use of such structures.

In other words, FTR tends to entangle the notional domains of time and probability, and both domains impact subjective estimations of value. Research which isolates only one of these factors (time) may be producing biased results due to unmeasured confounding variables (probability). Alternatively, the grammaticization of modality may actually be driving reported results. At the same time, the extent to which the encoding of future probability is obligatory in strong‐FTR languages is not known (as far as we know). As we have pointed out, modal systems are flexible enough to permit lexical workarounds. Additionally, arguments among linguists have not resolved questions as to the modal semantics of future tenses, despite this having implications for the linguistic savings hypothesis. Therefore, these factors should be studied in a sample of both weak‐ and strong‐FTR languages. This is what we undertook to do.

### 1.4 Study overview and hypotheses

To establish FTR language use, we created an FTR‐elicitation task based on Dahl's (1985, 2000b) FTR questionnaires. K. Chen's (2013) FTR status dichotomy is largely based on work by Dahl and colleagues in the EUROTYP Working Group on Tense and Aspect (Dahl, 2000a), so this was an appropriate starting point. In this task, participants were given a context and a target sentence. The main verb in the target sentence was unconjugated, and participants were asked to render the target sentence given the context. All contexts referred to future events. We made several modifications to the original questionnaire. In addition to creating many new items, we modified the contexts to include information of the likelihood of the referenced event occurring. This change was made in order to elicit modal future‐referring language. We refer to this as the “modality condition.” In order to elicit language from a wide variety of contexts, we included a range of temporal distances from time of speech as well as examples from each FTR mode.

After completing the FTR‐elicitation task, participants completed two additional measures which allowed us to establish whether future tenses encode temporal or modal notions. In the first instance, participants rated FTR structures in terms of whether they perceived them to be temporally distal or temporally proximal. In the second, they rated FTR structures in terms of whether they perceived them to encode high or low certainty. We made several predictions.

#### Predictions about FTR mode:

A modal verb is obliged in prediction‐based FTR in English but not Dutch, and most modals are low certainty (see Section 1.2.2). We, therefore, predicted that English—but not Dutch—speakers would be more likely to use low‐certainty language for prediction‐based FTR. We refer to this as the uncertain predictions hypothesis.

#### Predictions about modality condition:

Relativity researchers have postulated that the grammatical obligation to mark some domain can, over time, cause speakers to become more attentive to that domain (Wolff & Holmes, 2011). If English obliges speakers to encode notions of low‐certainty FTR, we reasoned that this might make speakers more attentive to the modal characteriztics of the speech context. We, therefore, predicted that use of low‐certainty language would be higher for English participants in the low‐certainty condition. We refer to this as the low‐certainty‐sensitivity hypothesis, that is, because English speakers are predicted to be more sensitive to the low‐certainty condition.

#### Predictions about effects of temporal distance in the FTR‐elicitation task:

We reasoned that if English speakers use more low‐certainty FTR language, this could, over time, lead to stronger cross‐modal mapping between temporal distance and notions of low certainty, that is, that English speakers might construe temporally distant events as inherently uncertain. We, therefore, predicted that English speakers would use more low‐certainty language as a function of temporal distance, but this would not be true of Dutch speakers. We refer to this as the English cross‐modal‐mapping hypothesis.

#### Predictions about temporal‐distance ratings:

We made two predictions about temporal‐distance ratings. On the basis of the linguistic savings hypothesis, we predicted that (a) future tenses would be rated as more distant than present tenses and (b) that Dutch participants would construe future events as more proximal (since higher future tense use in English should lead speakers to construe future events as distal). We refer to these predictions as the linguistic‐savings‐distance hypotheses.

#### Exploratory analyses:

With regard to ratings of high versus low certainty, we did not make any hypotheses. Rather, we chose to conduct exploratory analyses.

## 2 Methods

### 2.1 Participants

A final sample of N=651 participants completed the study (n=330 in (British) English [n=165 female, n=162 male, n=3 other], n=321 in Dutch [n=161 female, n=159 male, n=1 other]). This is after one participant was excluded because their age datum was missing. Data were collected between September and November 2019. English participants were recruited from Prolific Academic and Dutch participants were recruited from Qualtrics. Participants were native English and Dutch speakers currently residing in the United Kingdom and the Netherlands. The sample was matched to United Kingdom population norms for age and sex. Ethical approval for the study was granted by the University of Oxford Internal Review Board (ref. no. R39324/RE001). All participants were remunerated.

### 2.2 Materials

The study comprised three tasks: (1) an FTR‐elicitation task designed to establish future‐referring language, (2) a subjective‐temporal‐distance task designed to establish whether the tense of an FTR statement (future vs. present) impacted participants' construals of future temporal distance, and (3) a subjective‐certainty task designed to establish whether participants construed FTR structures as encoding high or low certainty.

#### 2.2.1 The FTR‐elicitation task

Participants were given a context and a target sentence and were tasked with typing in the conjugated target sentence. Before starting, participants were advised that there “were no correct answers,” and that they should complete the questionnaire sentences, “as though they were speaking to a close friend.” They were given two training items with example responses, and one trial item where they typed in a response. These were in the past tense in order to avoid biasing participants. There was one attention check: At a random point, participants were instructed to enter the word “dance” (Dutch “dans”). If they failed to do this, there were ejected from the survey immediately.

There were three within‐subjects factors in the task: FTR mode (predictions, intentions, schedules; modality condition (high certainty , low certainty, neutral); and temporal distance (1 month, 2 months, 3 months, 6 months, 1 year, 5 years). FTR mode was operationalized by constructing contexts which matched the criteria given in Section 1.2.3. Temporal distance was operationalized using temporal adverbials in the contexts, for example, “1 month,” “1 weeks,” etc. Modality condition was operationalized by giving participants numerical “certainty information” above each target sentence, for example:

**Context**: Chris's brother {SEND} him some money next month. You never know with him… When he gets it…
**Certainty**: 50% certain.
**Target**: …he {SPEND} it at the bar.

A typical response might be “He'll likely spend it at the bar.” Prior to starting, participants were told “there will be some ‘certainty information’ included in the context.” They were informed that “this indicates how certain you are about what you are saying.” They were then directed to “please imagine you are this certain and write down what you would say.” For schedules and predictions, participants were told they were supposed to be “___% certain”, and for intentions they were told they were supposed to be “___% decided” (this was because it was difficult to make “certain” agree with all intention contexts). In the low‐certainty condition, certainty information varied between 40%, 50%, and 60%. This was implemented to try to maintain participant engagement. In the high‐certainty condition, certainty information was invariably 100%. In the neutral condition, no certainty information was given. In creating FTR mode, we counted as intention any intention statement whether it was first or third person. This was to try to isolate second‐person intention (which can be difficult to differentiate from prediction, for example, *John will go out later*) from language usage in more prototypical prediction contexts.

The modality conditions were constructed by conserving syntactic structure while minimally altering semantic details between items at matched levels of temporal distance and FTR mode. This was done in order to address the possibility that idiosyncratic aspects of items were driving language usage. Semantic details (nouns, names, pronouns) were altered, but other linguistic details (e.g., sentence length and syntactical structure) were only minimally changed to ensure the certainty information did not clash with the certainty implied by the context of the item (see Table 1 and Supporting Information Figs. A.3 and A.4; see the Supporting Information for full questionnaire and example responses).

In each temporal distance by modality condition, there were five critical items: three prediction items, one intention item, and one scheduling item. This means there were 15 critical items per temporal distance, $3_{prediction}\times 3_{mod.cond.}+1_{intention}\times 3_{mod.cond.}+1_{schedule}\times 3_{mod.cond.}=15$. There were 90 critical items in total, $6_{temp.dist}\times 5_{FTRmode}\times 3_{mod.cond.}$. Because of time constraints, each participant completed 60 randomly selected trials. Trial order was randomized, and one trial was displayed per page.

**Table 1:** Example of the FTR‐elicitation task conditions

Modality Condition	FTR Mode	Certainty Information	Item
High certainty	Intention	100% decided	[Jen's uncle {SEND} her some money next month. She just loves skiing. When she gets it…] …she {BUY} a new pair of skis.
	Prediction	100% certain	[A to B: Don't invest in commodities. The market is very shaky…] …it {CRASH} within a month.
	Scheduling	100% certain	[In June: Q: When do you and Jen fly to France? A: She {SAID} it {BE} the 17th, but I just {CHECK} the schedule…] …we {LEAVE} on the 15th.
Neutral	Intention	‐	[Chris's father {SEND} him some money next month. When he {GET} it…] …he {BUY} a present for Amelie.
	Prediction	‐	[A to B: Don't invest in derivatives. The market is fraudulent…] …It {CRASH} within a month.
	Scheduling	‐	[In November: Q: When do you fly to Mexico?] A: My flight {LEAVE} on 15 December!
Low certainty	Intention	50% certain	[Chris's brother {SEND} him some money next month. You never know with him. When he gets it…] …he {SPEND} it at the bar.
	Prediction	40% certain	[A to B: Don't invest in Latin America. Conditions are unstable…] …Brazil {CRASH} within a month.
	Scheduling	60% decided	[In February: Q: When do you fly to Spain? A: I have to check the ticket…] …my flight {LEAVE} 15 March.

*Note*. Minimal alterations between modality conditions were implemented to constrain possible idiosyncratic item effects (due to irreconcilable semantic differences across FTR modes and temporal distances, this was not possible across the levels of these conditions). For example, the intention item in each certainty condition follows the conserved structure: “[PERSON]'s [FAMILY MEMBER] send [PRONOUN] some money next month. When [PRONOUN] {GET} it [PRONOUN] [VERB] [NOUN PHRASE].” In this case, and in others, the low‐certainty condition was minimally modified further.

##### Text classification:

After an initial exclusion of n=240 observations because of missing demographic data, there were N=38,398 text responses. It was therefore necessary to automate the scoring of responses in terms of whether they used the present tense, future tense, or some kind of modal expression. To accomplish this, we wrote a keyword‐based, deterministic, closed‐vocabulary classification program written in Python Python Software Foundation (2017). We refer to it as the FTR‐type classifier. It comprises a number of word lists which are used in combination with a set of rules to classify text items according to which tense and/or modality words they contain. The FTR‐type classifier categorizes text data into four exclusive semantic categories: future tense, present tense, low certainty, and high certainty. The latter two are further divided into two non‐exclusive categories based on whether a modal verb or some other construction type is used (see below). Each category is coded with (1) to indicate a response is a positive example, otherwise (0). These comprise the dependent variables for this task.

In English and Dutch, modal words can be used in combination with the future and present tense. For example, *It will probably rain* and *It will definitely rain* are both future tense, but different epistemic commitments are expressed. Similarly, *They could win tonight* and *The game definitely is at 7* are both present tense, but different modal notions are expressed (on present time modals see Condoravdi, 2002). Since we were not interested in formal tense structure and were rather attempting to explore differences in marking of the notional domains involved, it was appropriate to have epistemic modal morphemes “dominate” tense morphemes. Specifically, responses which used both tense and modal words were classed as low certainty (or high certainty) and not also as future or present tense. We outline the FTR‐type classifier categories classification system below (see the Supporting Information).

present tense: Responses were classed as present tense if they conjugated the main verb in the target sentence using the present tense and also failed to be classed as any of the other categories.

future tense: Responses were classed as future tense if they used commonly accepted “future” auxiliaries or explicit temporal adverbials (English *will, shall, be going to, about to*; Dutch *zullen* ‘will,’ *gaan* ‘be going to,’ *staat op* ‘about to’). Any response exhibiting these words, without additional modal epistemic words, was counted as future tense.

verbal‐low‐certainty: Responses which used low‐certainty modal verbs were classed as verbal‐low‐certainty (English *can, could, may, might, should*; Dutch *kunnen* ‘may’). A prototypical example is *This team might/may/could/should win tonight*.

verbal‐high‐certainty: Responses which used modal verbs which encode high certainty were classed as verbal‐high‐certainty (English *must*; Dutch *moeten* ‘must’). A prototypical example is *I must remember to take in the laundry*, although this suggests a deontic or bouletic base (i.e., having to do with obligations or desires, respectively). In fact, clearly epistemic contexts in which *must* sounds natural in English are difficult to find, for example, *The test tonight must be difficult* seems to again suggest a bouletic rather than epistemic base. We nonetheless include it as the only criteria for the verbal‐high‐certainty category.

other‐low‐certainty: Responses which used modal expressions indicating low certainty (apart from modal verbs) were classed as other‐low‐certainty. This includes low‐certainty modal modifiers (English *possibly, probably, potentially*, etc.; Dutch *misschien* ‘perhaps,’ *mogelijk* ‘possibly,’ *waarschijnlijk* ‘probably,’ *wellicht* ‘maybe,’ etc.). A prototypical example of a modal modifier encoding low‐certainty FTR is *It will possibly rain tonight*. It also includes low‐certainty mental state predicates (English *think, believe, reckon*, etc.; Dutch *denken* ‘think,’ *annehm* ‘assume,’ *veronderstellen* ‘suppose,’ etc.). A prototypical example might be, *I think it's going to be a hard win*. Finally, it also includes low‐certainty epistemic modal particles (Dutch *wel eens, wel*, approximately ‘well be,’ ‘well,’ as in *There could well be rain later*.).

other‐high‐certainty: Responses which used modal expressions which encode high certainty (apart from modal verbs) were classed as other‐high‐certainty. This includes modal modifiers (English *certainly, definitely, absolutely*, etc.; Dutch *zecker* ‘certainly,’ *definitief* ‘definitely,’ etc.). A prototypical example is *The storm will definitely hit the east coast this week*. It also includes high‐certainty modal particles (Dutch *toch*, approximately ‘fixed,’ ‘firm’).

##### Data exclusions:

The FTR‐type classifier cannot accurately classify responses which use negations, or responses which use words from two conflicting class criteria keyword lists. We refer to these as “mixed modal” responses. In the first instance, modal keywords switch polarity in the presence of negations. For instance, *I'm not certain it will rain tomorrow*, expresses low certainty. However, because of the presence of the high‐certainty class‐criterion keyword *certain*, it would be classed as high certainty. Similar in‐determinability characterizes mixed modal responses. For instance, *Rain tomorrow is certainly possible* expresses moderate certainty, but would be classed as both other‐high‐certainty and other‐low‐certainty because of the present of the class‐criterion keywords *certainly* and *possible*. Since such responses were in practice low frequency, our strategy was simply to exclude them from data analysis. We, therefore, detected the presence of negations using an averaged perceptron tagger following Collins (2002) but with Brown cluster features as described by Koo, Carreras, and Collins (2008) and using greedy decoding (implemented in *spaCy*; Explosion AI, 2020). Of the total responses, n=471 were excluded (n=191 mixed‐modality responses, n=229 negations, and n=51 because they were in both of these categories). This left a final sample of n=37,927 responses.

##### FTR‐type classifier reliability testing:

To test the reliability of the FTR‐type classifier, linguistically trained coders annotated N=1006 responses (n=504 in English, and n=501 in Dutch). Where systematic errors were found, the FTR‐type classifier was adjusted. After this process, all accuracy metrics were >0.99 (see the Supporting Information).

#### 2.2.2 The subjective‐temporal‐distance task

In this task, participants were given two phrases. One used the future tense (English “Ellie *will* arrive later on”; Dutch “Ellie *zal* later aankomen.”), and the other used the present tense (English “John *is arriving* later on”; Dutch “John *arriveert* later”). We refer to this manipulation as “tense condition.” Both used the temporal adverbial “later on” to ensure that participants construed the present tense frame as referring to the future. Participants rated subjective temporal distance using a slider between “close to now” (0) and “far from now” (10). Numbered slider intervals were not displayed. Prior to starting, participants were told “you will also be asked to indicate how far away from you a length of time feels.” For each item, they were told to “Indicate with the slider how far away from NOW the given time feels to you.” Before beginning, participants were given one example involving past time reference (“9 months ago”). As a distraction task, participants also rated 10 objective future distances (later today, 1 week, 1 month, 2 months, 3 months, 6 months, 9 months, 1 year, 2 years, and 5 years). Item order was randomized, and one item was displayed per page.

#### 2.2.3 The subjective‐certainty task

In this task, participants used a slider to rate between “uncertain” (0) and “certain” (100) how much certainty they construed a given FTR statement as expressing. FTR statements were created imputing different common FTR constructions types into the same “base” sentence: “It {RAIN} next week.” We chose representative examples from each of the coding categories of the FTR‐type classifier: future tense (“It will rain…”), present tense (“It is raining…”),^6^ verbal‐low‐certainty (“It could rain”), other‐low‐certainty (“It will possibly rain…”), and other‐high‐certainty (“It will definitely rain…”). Verbal‐high‐certainty was excluded because *must/moeten* are used to express *deontic* notions rather than epistemic high certainty about the future (Nuyts, 2000). (For a complete set of the items, see Fig. 7). Prior to beginning, participants were told “you will be asked to indicate how much certainty each statement expresses in YOUR eyes.” For each item, they were told, “Indicate how much certainty YOU would be expressing in the following statement.” Before starting, they were given one training example involving past time reference: “I think Pete picked up bread yesterday.” Item order was randomized, and one item was displayed per page.

### 2.3 Procedure

The study was hosted on the Qualtrics survey platform and was conducted online. (Participants recruited on Prolific were linked through to the Qualtrics survey.) It had a mixed design. The within‐subjects factors for each task are described above. There was one between‐subjects factor: survey language. At the beginning of the surveys, participants confirmed their first language and current residence. English speakers confirmed they were native English speakers residing in the United Kingdom, and Dutch speakers confirmed they were native Dutch speakers residing in the Netherlands. If they did not, they were immediately ejected from the survey. Following this, they answered some demographic questions (age, sex, income, education, marital status, and employment status), which were recorded as control variables. To understand whether multilingualism was affecting language elicited language, participants then completed a second‐language proficiency measure, in which they self‐rated their proficiency for up to three second languages. Ratings were between “can ask directions and answer simple questions” (1) and “very fluent, can use the language as well as a native language” (5) (see the Supporting Information). Following this, participants completed the FTR‐elicitation task, the subjective‐temporal‐distance task, and then the subjective‐certainty task.

## 3 Results

**Fig. 2:**
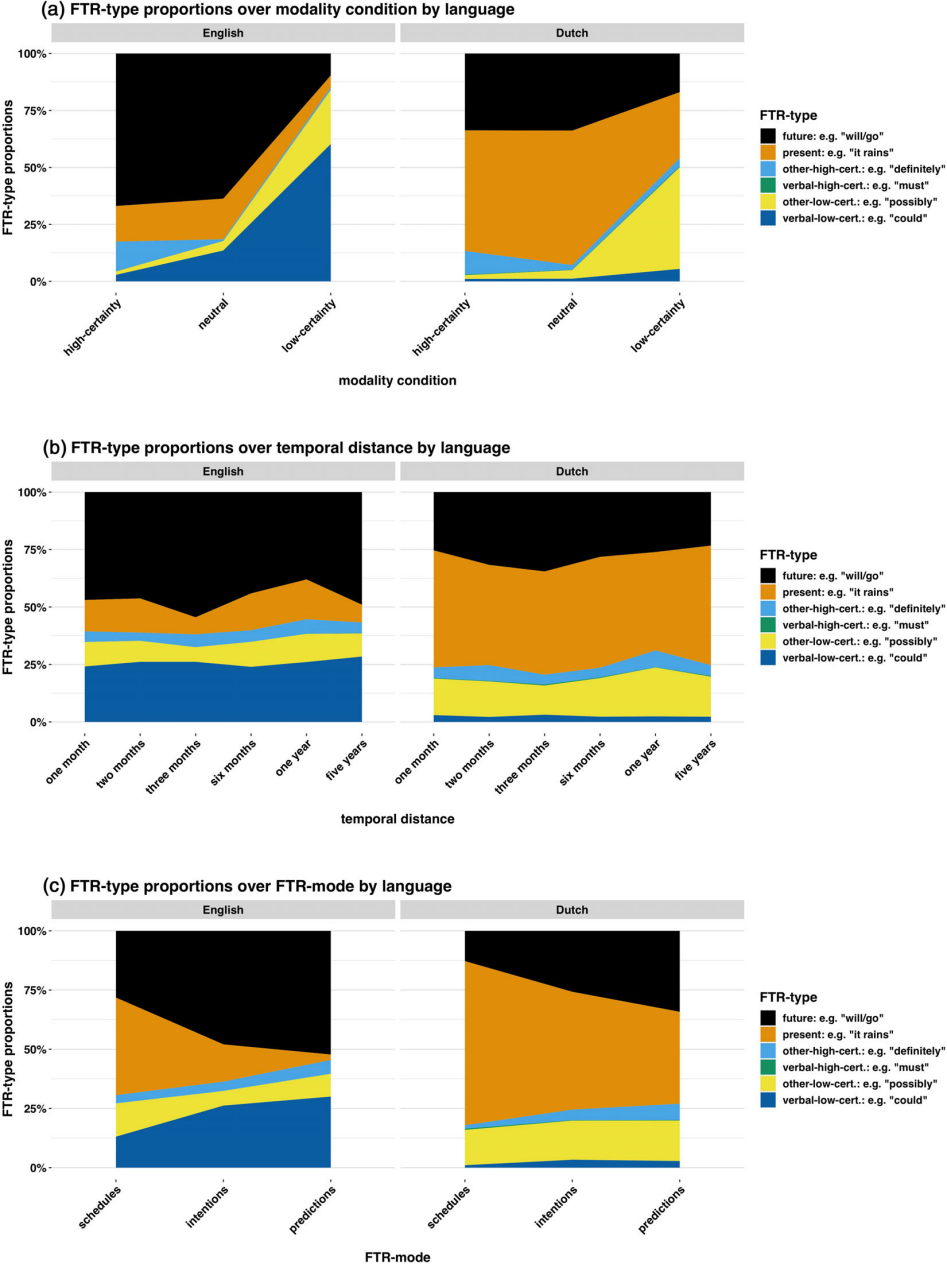
FTR‐type proportions over modality condition, temporal distance, and FTR mode. Dutch speakers used more present tense and fewer future tense constructions. English speakers use more low‐certainty modal verbs. Dutch speakers made up for this to some degree through the use of other low‐certainty constructions.

We present an overview of results in Fig. 2. English speakers used more future tense and fewer present tense constructions. This reflects well‐known differences between English and Dutch, that is, FTR status. Additionally, English speakers appeared to use more low‐certainty language than Dutch speakers. This was mostly driven by modal verb use, for example, *It could/may/might rain*. English speakers used more low‐certainty language for predictions than another other FTR mode, a pattern which did not characterize Dutch (Fig. 2c).

To test our hypotheses, we combined verbal‐low‐certainty and other‐low‐certainty into a single dichotomous variable (“low certainty”) which was (1) for any response which encoded low certainty and otherwise (0). For example, responses like *I think/believe/guess it will rain*, *It will possibly/probably/potentially*, and *It could/might/may/should/can rain* would all be classed as low certainty (1). Multilevel modeling was appropriate, as responses from a single participant were likely to be similar across different items, and responses to a single item were likely to be similar across different participants. We followed research practice by building models sequentially and using log‐likelihood ratio tests to ascertain whether adding variables improved model fit (Aguinis, Gottfredson, & Culpepper, 2013; Legler & Roback, 2019; Raudenbush & Bryk, 2002; Twisk, 2006). Using generalized linear regression with a logit link function (logistic regression), we regressed binary (0,1) low‐certainty language over a fixed intercept and then allowed intercepts to randomly vary by item and participant. We added fixed effects for language, FTR mode, modality condition, and temporal distance. For temporal distance, we used the natural log of the number of days from time of speech. Since effects might be expected to vary interactively, we also included all two‐way interactions between these variables. Finally, we allowed slopes for language to vary by item, which allowed us to statistically capture differences in the random effects of items in both languages. All of these steps were significant, p<.001. By modeling such variance, we were able to estimate parameters of fixed effects independently of item‐by‐language‐level and participant‐level idiosyncrasies. Inspection of random effect, plots over normal quartiles indicated estimate bias was within tolerable bounds (Maas & Hox, 2004) (the Supporting Information). Some demographic variables significantly predicted low‐certainty language use. We included these (see Table 2). We also included effects of item order, which was significant (the Supporting Information).

**Table 2:** Low‐certainty language use regression coefficients

	$e^{\beta }$	SE	*z*‐Score	*p*
(Intercept)	0.18	0.12	−13.91	<.001${}^{***}$
Language: Dutch	0.24	0.14	−10.2	<.001${}^{***}$
FTR mode: prediction	2.56	0.07	13.21	<.001${}^{***}$
FTR mode: intention	0.9	0.09	−1.18	.237
Modality condition: high certainty	0.09	0.1	−24.06	<.001${}^{***}$
Modality condition: low certainty	28.79	0.08	41.7	<.001${}^{***}$
Temporal distance	1.08	0.05	1.79	.074^·^
Item order	1.06	0.02	2.66	.008${}^{**}$
Language: Dutch * FTR mode: prediction	0.7	0.1	−3.61	<.001${}^{***}$
Language: Dutch * FTR mode: intention	1.16	0.12	1.21	.228
Language: Dutch * modality condition: high certainty	2.08	0.11	6.8	<.001${}^{***}$
Language: Dutch * modality condition: low certainty	0.72	0.09	−3.59	<.001${}^{***}$
FTR mode: prediction * modality condition: high certainty	1.19	0.11	1.64	.102
FTR mode: intention * modality condition: high certainty	0.9	0.14	−0.7	.487
FTR mode: prediction * modality condition: low certainty	0.5	0.09	−7.77	<.001${}^{***}$
FTR mode: intention * modality condition: low certainty	1.19	0.11	1.49	.137
Language: Dutch * temporal distance	0.98	0.06	−0.24	.807
Modality condition: high certainty * temporal distance	0.86	0.07	−2.13	.033*
Modality condition: low certainty * temporal distance	1.06	0.06	0.95	.345
Language: Dutch * item order	0.86	0.04	−4.29	<.001${}^{***}$
Age	1.15	0.07	2.2	.028*
Employment: employed	0.83	0.1	−1.93	.054^·^
Employment: student	1.22	0.18	1.12	.264

*Note*. Coefficients are exponentiated, so represent changes in the odds ratio of using a low‐certainty term. Age was mean centered at 0 and scaled such that SD=1. Modality condition, FTR mode, and employment were sum‐coded, so coefficients represent level‐wise differences from the grand mean, and interactions can be interpreted as marginal effects with variables at mean. Only those demographics the addition of which improved model fit were included, p<.05. We also tested whether multilingualism affected elicited language. We operationalized this as $S_{i}=\sum_{k}p$, where *S* is the sum for participant *i*, of self‐reported proficiency *p* (1–5) for up to *k* (0–3) second languages. In no case did adding this improve model fit, p>.1. Generally, English speakers used more future and low‐certainty constructions as the task progressed, and Dutch speakers used more present constructions, suggesting speakers trended towards to language‐level norms as they progressed. For random components see the Supporting Information. ${}^{***}p<.001$; ${}^{**}p<.01$; ${}^{*}p<.05$; ·p<.1

### 3.1 The uncertain predictions hypothesis

**Fig. 3:**
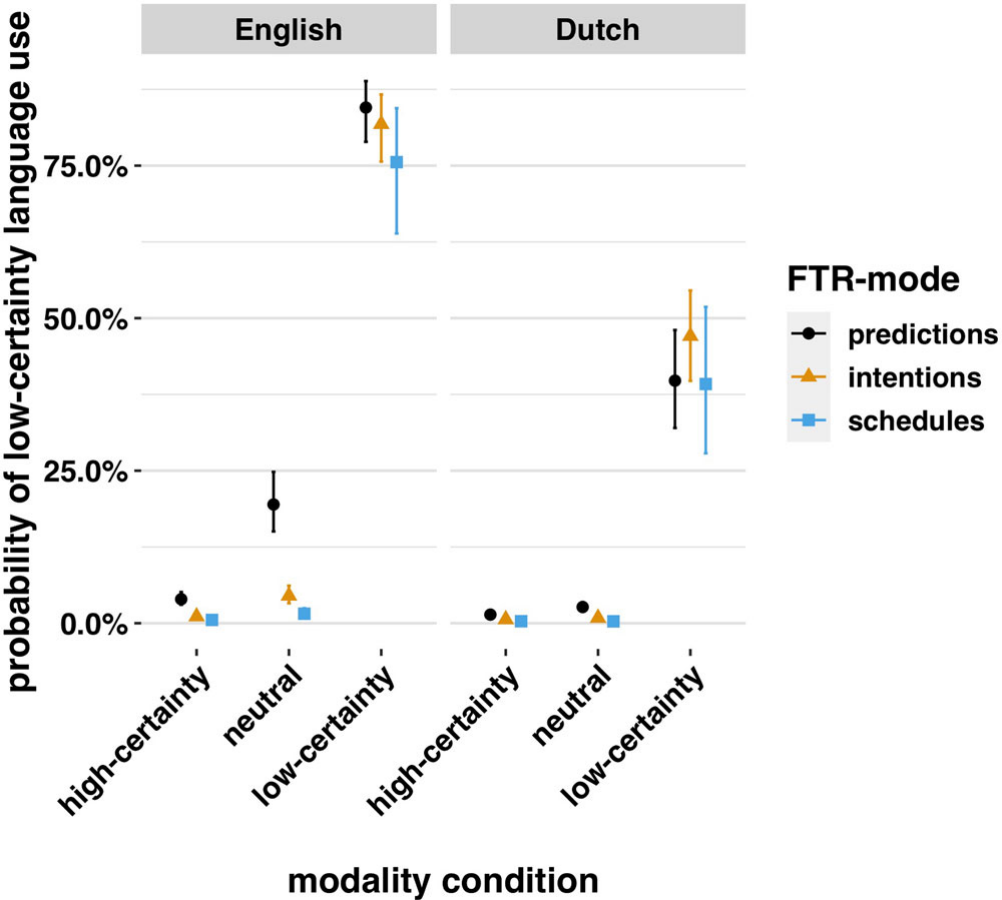
Low‐certainty language use over modality condition by FTR mode. Confidence intervals here and for Figs. 4, 5, 7, and 6 are calculated using the *R* package *ggpredict* by matrix‐multiplying a predictor *X* by the parameter vector *B* to get the predictions, then extracting the variance–covariance matrix *V* of the parameters and computing *XVX*’ to get the variance–covariance matrix of the predictions. The square root of the diagonal of this matrix represents the standard errors of the predictions, which are then multiplied by ±1.96 for the confidence intervals (Lüdecke, 2019). English speakers used more low‐certainty constructions, particularly when making predictions in the neutral condition and in the low‐certainty condition overall.

We had predicted that relative to intentions and schedules, English speakers would use more low‐certainty terms when making predictions. We predicted that this would not be the case for Dutch speakers. To test this, we used the *emmeans* package to conduct planned comparisons for the effect of FTR mode by language averaged across modality condition. We found that compared with intentions, English speakers used significantly more low‐certainty constructions when making predictions, $e^{\beta }=2.86,\;SE=0.13,\;z=8.1,\mathrm{and}\;p<.001$. Contrary to our prediction, we found that Dutch speakers did as well, $e^{\beta }=1.75,\;SE=0.19,\;z=3,\mathrm{and}\;p=.032$. However, they did this to a much lesser extent and inspection of Fig. 3 suggests significant effects were driven by high model confidence around low‐frequency low‐certainty language use in the certain and neutral conditions. Indeed, Dutch speakers making predictions used significantly fewer low‐certainty constructions than English speakers, $e^{\beta }=0.17,\;SE=0.14,\;z=-13.08,\mathrm{and}\;p<.001$. A particularly striking effect is that English speakers used low‐certainty language when they made predictions in the neutral condition (Fig. 3). This pattern is not evident in the Dutch data. These results support the uncertain predictions hypothesis. They suggest that the grammaticization of FTR may involve increasing obligatorization of the encoding of low certainty when making predictions.

### 3.2 The low‐certainty‐sensitivity hypothesis

Next we wanted to understand effects of modality condition. We had predicted that English speakers would be more sensitive to modality condition, using more low‐certainty language in the low‐certainty condition. As predicted, we found that English speakers were more sensitive to our certainty manipulation. Averaged across FTR mode, English speakers in the low‐certainty condition used significantly more low‐certainty language than Dutch speakers did, $e^{\beta }=5.87,\;SE=0.15,\;z=11.86,\mathrm{and}\;p<.001$ (see Fig. 3). This indicates that in addition to using more low‐certainty language generally, English speakers used more low‐certainty language as a function of the low‐certainty condition. They were more sensitive to our manipulation of certainty.

**Fig. 4:**
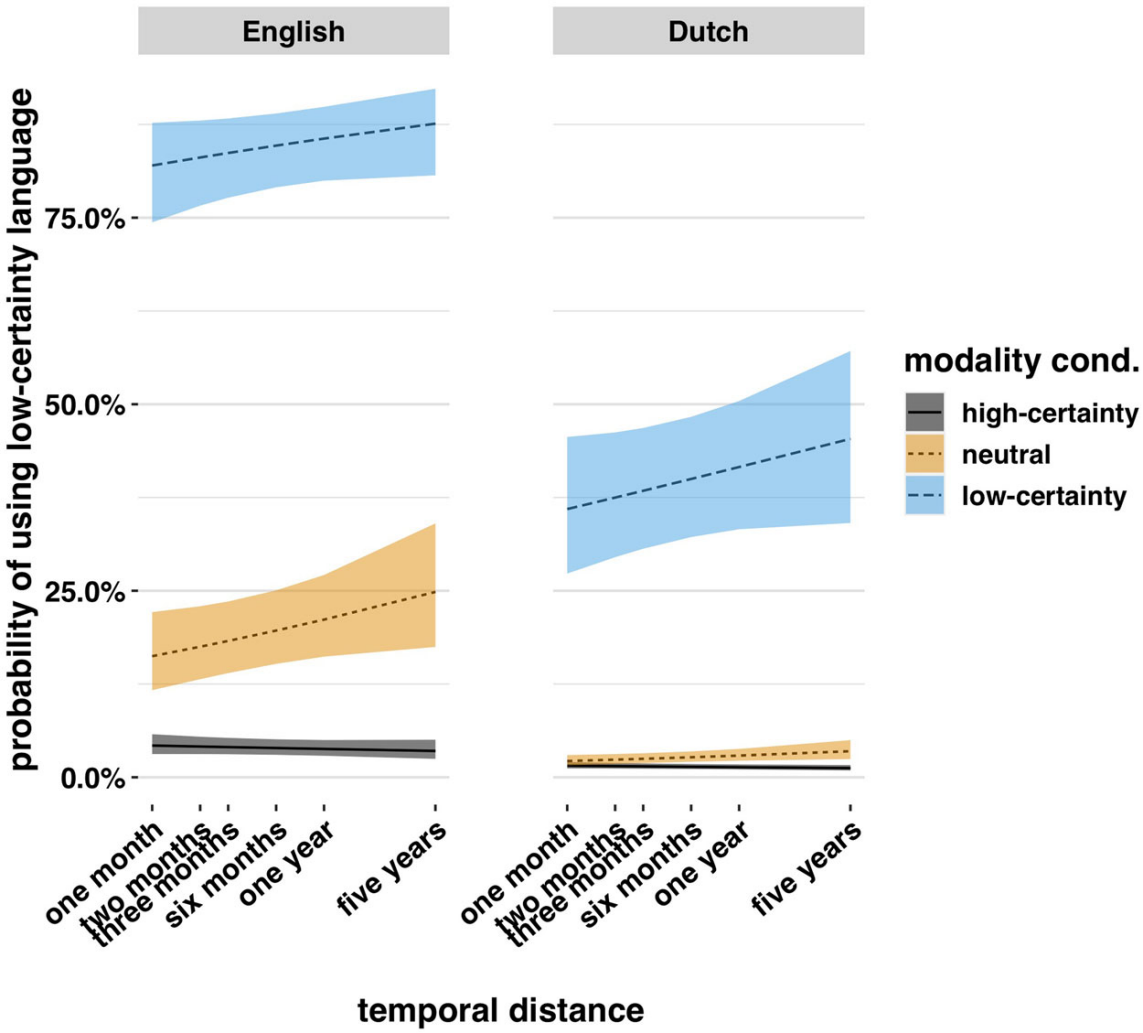
Low‐certainty language use over temporal distance by modality condition. In the low certainty and neutral conditions, English speakers used more low‐certainty language as temporal distance increased. Dutch speakers did not. The *x*‐axis is log‐scaled.

### 3.3 The English cross‐modal‐mapping hypothesis

Next we wanted to understand how temporal distance impacted low‐certainty language use. We had predicted that English—but not Dutch—speakers would use more low‐certainty language as a function of temporal distance.

To test this hypothesis, we estimated the slope for uncertain language use over temporal distance in the neutral modality condition (since the hypothesis posits that temporal distance will be cross‐modally mapped onto notions of uncertainty in English, it would not make sense to test it in modality conditions which primed modal notions). As predicted, we found that English speakers used more uncertain language as a function of temporal distance in the neutral condition, $e^{\beta }=1.19,\;SE=0.08,\;z=2.29,\mathrm{and}\;p=.022$ (see Fig. 4).

Was the pattern in Dutch different? It was. In Dutch, the slope for low‐certainty language over temporal distance in the neutral condition was not significant, $e^{\beta }=1.17,\;SE=0.09,\;z=1.68,\mathrm{and}\;p=.093$.

These results support the English cross‐modal‐mapping hypothesis. English speakers used more low‐certainty language in the neutral and low‐certainty conditions as temporal distance increased. Dutch speakers did not.

### 3.4 The linguistic‐savings‐distance hypotheses

On the basis of the linguistic savings hypothesis, we had predicted (a) that participants would rate the future tense frame, “Ellie *will* arrive later on,” as more temporally distal than the present tense frame, “John *is arriving* later on”; and (b) that Dutch participants would rate the future as more temporally proximal than English participants. To test these predictions, we regressed subjective distance ratings over language and tense condition and the interaction between them. We used a multilevel linear regression with random intercepts for participant (these were significant, $\chi^{2}\left(1\right)=388.44,\;p<.001$).

**Fig. 5:**
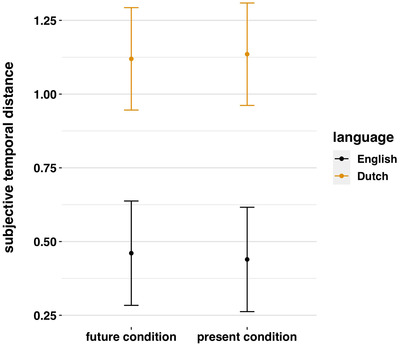
Effect of tense condition on subjective temporal distance ratings in speakers of English and Dutch.

Was the future tense frame construed as more distant? It was not (Fig. 5). Tense frame had no significant effect in either English, β=−0.02,SE=0.07,t(648)=−0.29,andp=.773, or Dutch, β=0.02,SE=0.07,t(648)=0.21,andp=.833. This indicates that participants were not construing the future tense statement as more temporally distal.

**Fig. 6:**
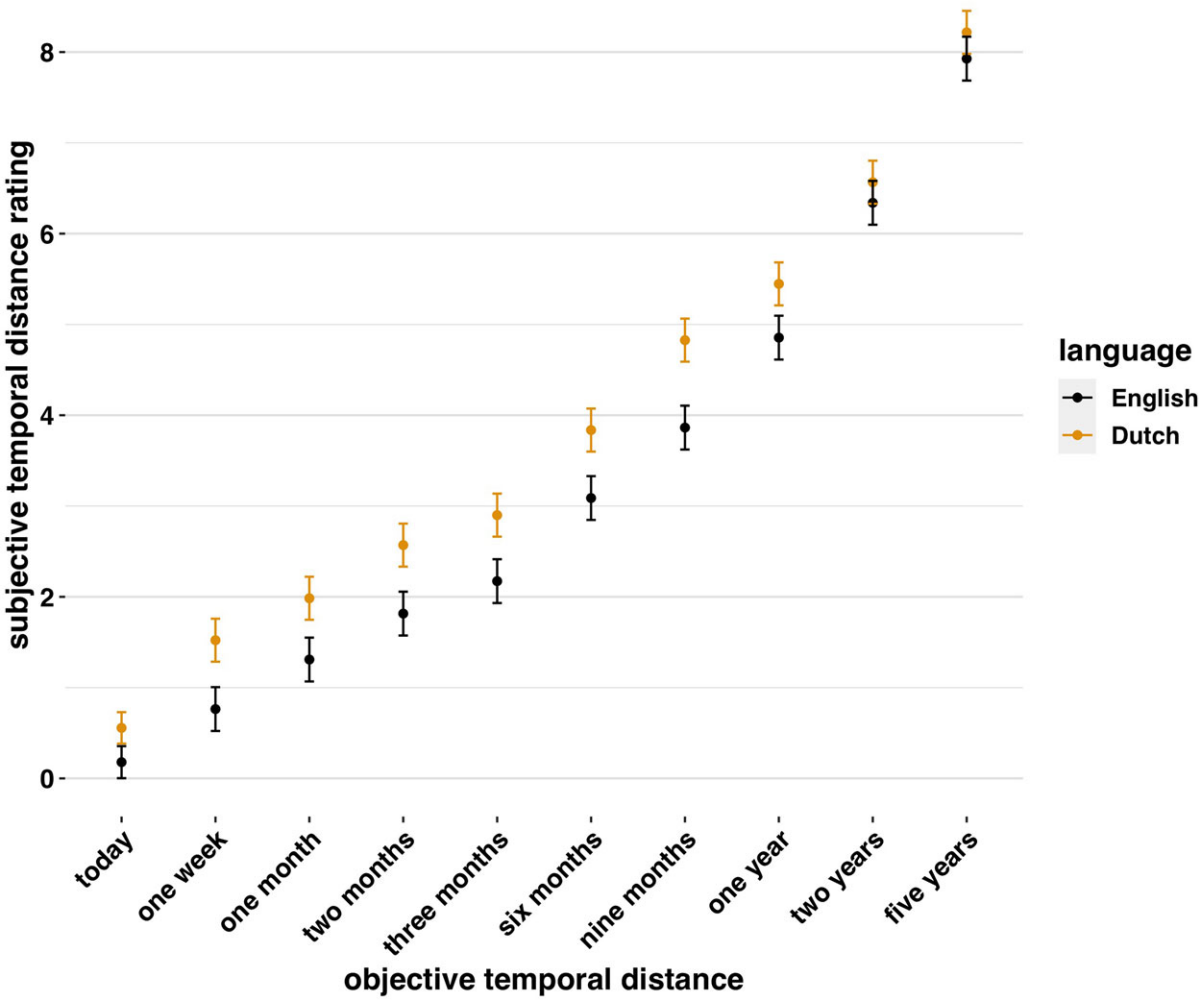
Subjective ratings of temporal distance by language and objective temporal distance.

Did Dutch speakers construe the future more temporally proximal? They did not. In fact, relative to English speakers, Dutch speakers rated the future as more distal (Fig. 5), and significantly so, β=0.66,SE=0.13,t(905.25)=5.21,andp<.001. This effect might have been limited to the temporal distance of the two “future/present” items (i.e., “later on”). To test whether, it was, we re‐estimated the model but using the objective distances in the distractor tasks, ranging from “later today” to “5 years.” We again found that Dutch speakers rated the future as more distal, β=0.61,SE=0.13,t(648)=4.84,andp<.001. This was particularly marked in temporal distances between 1 week and 1 year (Fig. 6). This is the opposite to the direction predicted by the linguistic savings hypothesis.

Together, these results fail to support the hypothesis that tense framing impacts construals of temporal distance and that therefore Dutch speakers construe the future as closer in time (cf. Chen, 2013).

### 3.5 Exploratory analyses: The subjective probability task

**Fig. 7:**
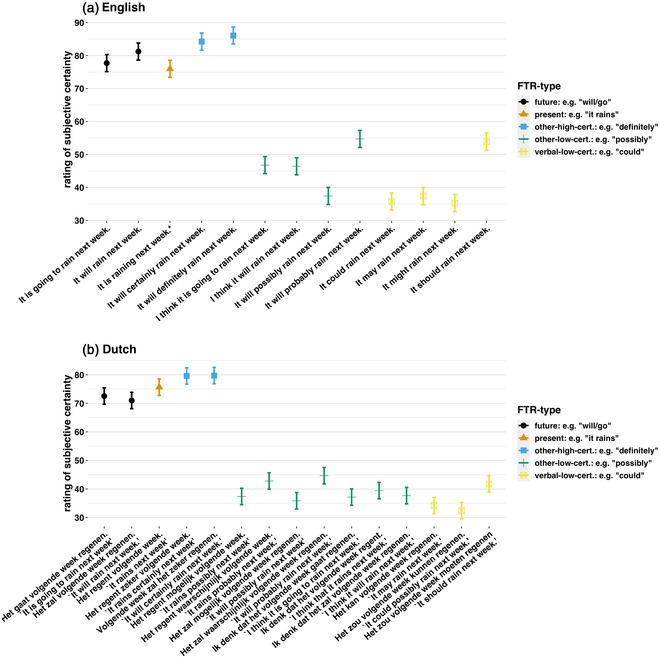
Subjective ratings of certainty by item and FTR type in English and Dutch. In both languages, future and present tense appear to encode high certainty, while modal and other‐low‐certainty constructions encode low certainty. English speakers appeared to break modal polarity into finer gradations than Dutch speakers, with clearer differences between low certainty (*could/may/might*) and intermediate‐certainty (*should/probably/I think*) modal expressions. * We acknowledge that present tense prediction (*It is raining…*) is either low‐frequency or unacceptable in English. We nonetheless included this item to maintain comparability with Dutch data. Certainty ratings for this item should be interpreted as speculative.

To explore the results of the subjective probability task, we regressed certainty ratings over an unordered factor which indexed each item. Because the items were not strictly comparable between English and Dutch, we did this separately for each language. We included random intercepts for participant. This was significant in both languages, p<.001. We present the results in Fig. 7. We were particularly interested in the future tenses, given the conflicting accounts that they either encode modal strengthening or modal weakening. Interestingly, future tenses in both languages were rated as high certainty. This undermines accounts which suggest future tense marking encodes modal weakening.

However, the languages differed in subtle ways. In discussing these results, we will use the term “modal polarity” to refer to the one‐dimensional scale between high and low certainty. English appeared to break modal polarity into finer gradations, with clearer differences between low certainty (*could/may/might*) and intermediate certainty (*should/probably/I think*). This suggests that English may oblige speakers to express greater degrees of precision about the likelihood of future outcomes.

Additionally, we found that relative to the present tense, Dutch speakers rated the future tense as significantly less certain, β=−3.9,SE=1.43,t(4770)=−2.72,andp=.007, and English speakers rated it as significantly more certain, β=3.5,SE=1.43,t(3960)=2.44,andp=.015.

## 4 Discussion

The study supports the hypothesis that the encoding of modality is implicated in FTR grammaticization processes. We found that English speakers were more likely to mark their predictions using an low‐certainty construction. English speakers also used more low‐certainty language as a function of temporal distance. This suggests that English speakers construe temporally distant events as increasingly uncertain. Additionally, English speakers were more sensitive to modality condition. They used more low‐certainty language in the low‐certainty condition. All these results suggest that, relative to Dutch speakers, English speakers are more likely to encode low‐certainty notions when they talk about the future. The fact that it was mostly modal verbs which drove this effect (Fig. 2) suggests grammatical constraints are responsible.

In exploratory analyses of the subjective probability task, we found that both the future and present tenses were rated as high certainty. This suggests obliging their use would cause less, not more, discounting (cf. K. Chen, 2013). Additionally, differences in the relative modal polarity of the present and future tenses in English and Dutch suggest that FTR status may be a relevant determinant of modal future tense semantics.

Finally, we found no support for the account that the future tense encodes temporal distance. There was no difference in subjective‐temporal‐distance ratings as a function of whether a future‐referring statement was framed using the future or present tense. This suggests that the future tense does not encode temporal distance (cf. K. Chen, 2013). We also found that Dutch speakers rated future events as more distal than English speakers (cf. K. Chen, 2013). In combination, these findings suggest the temporal mechanisms hypothesized to underpin the relationship between FTR grammaticization and temporal discounting cannot be involved in producing observed effects—at least in English and Dutch.

### 4.1 FTR status: The weak/strong dichotomy

Do our results corroborate or undermine the FTR status dichotomy? English speakers used more future tense constructions (Fig. 2). However, they additionally used more low‐certainty language. Low‐certainty language use in English was also more sensitive to FTR mode, probability condition, and temporal distance. This was mostly driven by modal verbs, which means that grammatical features of English may be involved in producing higher encoding of low certainty, that is, the obligatory modal verb system.

This suggests that obligatory future tenses and stricter encoding of modality arise from a unified underlying process. Dahl (1985) delineated a “futureless area” comprising European languages which do not oblige the future tense for prediction‐based FTR. Obligatory tense marking in prediction‐based FTR is suggested to be reasonable proxy for FTR grammaticization in general. Our results suggest this includes the obligatorization of modal verbs as well as future tenses. As such, in one sense, the FTR status dichotomy was supported. FTR appeared more grammaticized in English, with the noted caveat that modal FTR structures were implicated in this difference.

An important point is that our results have no implications for whether it is *possible* to form nuanced linguistic FTR utterances in these languages. In pointing out that English speakers use more low‐certainty language, we do not imply that Dutch FTR is deficient, simple, or vague in what Dutch speakers *may* articulate. Our results rather suggest that English grammatical constraints nudge English speakers towards encoding more low‐certainty modality.

### 4.2 Future tense semantics: FTR status impacts tense semantics

In the subjective‐certainty task, we found that English speakers rated the future tense as highest certainty, while Dutch participants rated the present tense as highest. This finding supports our paradigmatic analysis. We pointed out above that in English the highest certainty FTR structure available for prediction‐based FTR is the future tense. In Dutch, present tense statements are possible. Our results are compatible with the conclusion that this difference causes differences in relative encoded certainty between the future and present tense in these languages. Moreover, the result suggests there are cross‐linguistic differences in the modal strength of future tenses and that FTR status is a determinant of these. This means obliging the use of the future tense is expected to affect psychological discounting differently in different languages.

### 4.3 Causal mechanisms: A modal account of observed findings

In a recent risky intertemporal‐choice task, Vanderveldt et al. (2015) found that a function of the following form best described empirical valuations of risky future rewards: $V=A/[{\left(1+kD\right)}^{sd}\times {\left(1+h\theta \right)}^{sp}]$. In this instance, *V* is monetary value, *D* is temporal distance, θ is odds against, *k* and *h* are parameters affecting the discounting rates, and sd and sp are scaling factors which have been found to best describe experimental evidence. This means that psychological discounting is better described by a discounting plane, than a discounting curve. Subjective value is a function of both the odds against and the time until the receipt of a future reward.

How might cross‐linguistic differences in FTR grammaticization impact such psychological discounting processes? First, the mechanisms proposed by the linguistic savings hypothesis might still be in effect. However, they could just as easily apply to probabilistic discounting, that is, we might predict speakers of languages which more strictly grammaticize FTR to both have relatively more precise beliefs about, and relatively lower estimates of, the probability of future events. We would thereby predict them to probabilistically discount more heavily. However, as the probability of a future reward decreases, temporal distance has an increasingly negligible effect on subjective value; in contrast probability discounting is relatively unaffected by temporal distance (Vanderveldt et al., 2015). We, therefore, suggest that differences in the grammaticization of probability (i.e., modality) may be the more important factor in driving observed cross‐cultural differences in discounting‐related behavior (cf. K. Chen, 2013). Real‐world (risky) intertemporal decisions could be impacted by such probability discounting differences. If the English case generalizes, this suggests that a “modal” account could plausibly explain many reported results (K. Chen, 2013; Chen et al., 2017; Chi et al., 2018; Figlio et al., 2016; Galor et al., 2016; Guin, 2017; Hübner & Vannoorenberghe, 2015b, 2015a; Liang et al., 2018; Lien & Zhang, 2020; Mavisakalyan et al., 2018; Pérez & Tavits, 2017; Roberts et al., 2015; Sutter et al., 2015; Thoma & Tytus, 2018).

### 4.4 Causal mechanisms: Temporal distance and precision

The results of the subjective‐temporal‐distance task did not support the linguistic savings hypothesis. English speakers rated future events as closer in time than Dutch speakers. This is the opposite to the direction expected if the English future tense encoded temporal distance. Additionally, we found that tense framing (future vs. present) had no effect on distance ratings. It is possible that this null result is an artifact of the single phrase we used: “___ *is arriving/will arrive* later on.” A difference might emerge with more distant FTR statements or other phrases. Future research could take this up. However, our findings are consistent with findings that tense framing does not affect intertemporal decisions. Banerjee and Urminsky (2017) conducted a series of six experiments investigating this. They had participants make intertemporal choices, which were framed in either the present or future tense, that is, “you get $10 in a week” versus “you *will* get $10 in a week.” In a series of several experiments which used a range of distances, such manipulations had no effect on participants' time preferences (a similar result is reported in Thoma & Tytus, 2018). This suggests that future tenses do not encode temporal distance, regardless of the temporal distances involved. Our findings corroborate this conclusion.

What do tenses encode? We found the present and future tenses were rated as high certainty in English and Dutch. This suggests obligatory future tense use would cause speakers to discount less, not more, the opposite to observed results. In fact, the ratio of high‐certainty (present + future + certain) versus low‐certainty language is the only linguistic feature we identified which might plausibly affect psychological discounting in the observed direction. This lends support to our general argument that FTR grammaticization impacts psychological discounting because it affects speakers beliefs about future risk rather than their construals of future temporal distance and/or precision.

### 4.5 Contributions to work on temporal‐distance representations

Dutch speakers rated the future as farther away. This contributes to a nascent body of literature which has begun to investigate how subjective ratings of future distance impact discounting (see Bradford, Dolan, & Galizzi, 2019; Kim & Zauberman, 2009; Zauberman, Kim, Malkoc, & Bettman, 2009). For instance, Thorstad and Wolff (2018) found that people whose tweets reference increasingly distant future times were more likely to invest in the future and less likely to undertake risky behavior. Ireland, Schwartz, Chen, Ungar, and Albarracín (2015) found that U.S. counties with higher rates of FTR tweets had lower rates of Human Immunodeficiency Virus (HIV). In this context, HIV exposure is expected to be impacted by time preferences because risky behaviors (e.g., intravenous drug use, unprotected intercourse) incur long terms costs (risk of contracting HIV) but confer short‐term benefits. Finally, using a measure similar to ours, Thorstad, Nie, and Wolff (2015) found that people who construed the future as farther away were more present oriented. Together, these results support K. Chen's (2013) proposal that subjective representational distance is a significant predictor of time preferences. However, we found that Dutch speakers represented the future as farther away. As far as we can tell, this is the first study to use time slider type tasks to identify cross‐cultural differences of this nature. If this is related to cross‐linguistic differences in FTR grammaticization, this suggests that higher obligation to mark future statements causes future events to be construed as more proximal by strong‐FTR speakers. However, if this were the case, it would cause strong‐FTR speakers to be more future‐oriented not less—as is hypothesized and observed. This entails that differences in construals of future distance are not likely to be causally implicated in the relationship between FTR grammaticization and psychological discounting.

### 4.6 Conclusions

In general, we found that FTR status indexes cross‐linguistic differences in the encoding of future modality. English speakers encoded low‐certainty modality more than Dutch speakers. This was mostly driven by a more highly grammaticized modal verb system. Moreover, we found that future tenses encode notions of high certainty, not temporal distance, or low certainty. This implies the effect of obligatory future tense marking would go in the opposite direction to that hypothesized by K. Chen (2013).

Together, these results undermine the notion that FTR grammaticization is primarily about time and call into question the validity of the causal mechanisms suggested in K. Chen (2013). If tense and modal FTR grammaticization are generally correlated, it may be the case that observed cross‐cultural differences in discounting‐related behavior actually involve probabilistic discounting driven by stricter encoding of modal notions in strong‐FTR languages.

Economists continuing to work on this question might begin exploring the complex potential relationships between FTR grammaticization and discounting. These processes are worth understanding: Psychological discounting processes are an important determinant of a wide range of behaviors, including health outcomes (Ireland et al., 2015; Vuchinich & Simpson, 1998), drug use (McKerchar & Renda, 2012), climate change attitudes (Mavisakalyan et al., 2018), educational performance (Figlio et al., 2016), pathological gambling (Hodgins & Engel, 2002), and investment in savings (Liu & Aaker, 2007). If the precise nature of the relationship between FTR grammaticization and discounting is better understood, researchers may be able to better understand how—or whether —cross‐linguistic differences impact the discounting mechanisms which underpin intertemporal decisions. Detailed experimental work which combines behavioral economic techniques with usage‐based typological linguistics should be employed to explore the precise relationships between cross‐linguistic differences in FTR grammaticization and psychological discounting.

## Notes

1 Typically, linguists use separate but related terms for notional categories and the linguistic structures which grammatically encode them (Bybee, Perkins, & Pagliuca, 1994). As such, we use “FTR” to refer to any statement about the future and “future tense” to refer to the linguistic structures which sometimes grammatically mark FTR, for example, in English *will*, *shall*, or *be going to*.

2 Dahl (2013) points out that these terms are problematic. FTR stands for “future time reference,” and, of course, it is possible in all languages to refer to future time (whether or not a tense is obliged). Better might be “strong‐FTR_G_” to indicate that the difference is a matter of Grammatical marking. However, the terms are in widespread use. We will not deviate from them, though wish to acknowledge Dahl's (2013) critique.

3 Negated high certainty (p=0) might also be added, for cases where a speaker is highly certain something is NOT the case.

4 Context is given in [brackets].

5 English uses the present progressive to refer to present time events. The zero‐form simple present tense, *It rains*, is used for gnomic statements, which have truth value independent of any deictic time reference, for example, *It rains in Oxford* (Broekhuis & Verkuyl, 2014).

6 We used the present progressive because the simple present tense *it rains* is not grammatical for English predictions.

## References

[cogs13224-bib-0001] Aguinis, H. , Gottfredson, R. , & Culpepper, S. (2013). Best‐practice recommendations for estimating cross‐level interaction effects using multilevel modeling. Journal of Management, 39(6), 1490–1528.

[cogs13224-bib-0002] Banerjee, A. , & Urminsky, O. (2017). What you are getting and what you will be getting: Testing whether verb tense affects intertemporal choices. Advances in Consumer Research, 45, 512–513. 10.1037/xge0001192 35286117

[cogs13224-bib-0003] Behydt, G. (2005). Future time reference—English and Dutch compared. In N. Delbecque , J. van der Auwera , & D. Geeraerts (Eds.), Perspectives on variation: Sociolinguistic, historical, comparitive, Trends in Linguistics. Studies and Monographs, volume 163 (pp. 251—‐274). Berlin, Germany: Mouton de Gruyter.

[cogs13224-bib-0004] Białaszek, W. , Ostaszewski, P. , Green, L. , & Myerson, J. (2019). On four types of devaluation of outcomes due to their costs: Delay, probability, effort, and social discounting. The Psychological Record, 69, 415–424. 32095026 10.1007/s40732-019-00340-x PMC7039538

[cogs13224-bib-0005] Bickel, W. K. , Jones, B. A. , Landes, R. D. , Christensen, D. R. , Jackson, L. , & Mancino, M. (2010). Hypothetical intertemporal choice and real economic behavior: Delay discounting predicts voucher redemptions during contingency‐management procedures. Experimental and Clinical Psychopharmacology, 18(6), 546–552. 21186929 10.1037/a0021739 PMC4034533

[cogs13224-bib-0006] Bickel, W. K. , Odum, A. L. , & Madden, G. J. (1999). Impulsivity and cigarette smoking: Delay discounting in current, never, and ex‐smokers. Psychopharmacology, 146(4), 447–454. 10550495 10.1007/pl00005490

[cogs13224-bib-0007] Bouma, L. (1975). On contrasting the semantics of the modal auxiliaries of German and English. Lingua, 37, 313–339.

[cogs13224-bib-0008] Bradford, W. D. , Dolan, P. , & Galizzi, M. M. (2019). Looking ahead: Subjective time perception and individual discounting. Journal of Risk and Uncertainty, 58, 43–69.

[cogs13224-bib-0009] Broekhuis, H. , & Verkuyl, H. J. (2014). Binary tense and modality. Natural Language and Linguistic Theory, 32(3), 973–1009.

[cogs13224-bib-0010] Bybee, J. , & Dahl, Ö . (1989). The creation of tense and aspect systems in the languages of the world. Studies in Language, 13(1), 51–103.

[cogs13224-bib-0011] Bybee, J. , Perkins, R. , & Pagliuca, W. (1994). The evolution of grammar. Chicago, IL: University of Chicago Press.

[cogs13224-bib-0012] Cariani, F. , & Santorio, P. (2018). *Will* done better: Selection semantics, future credence, and indeterminacy. Mind, 127(505), 129–165.

[cogs13224-bib-0013] Casasanto, D. (2016). Linguistic relativity. In N. Riemer (Ed. ), Routledge handbook of semantics (pp. 159–174). New York, NY: Routledge

[cogs13224-bib-0014] Chen, K. (2013). The effect of language on economic behavior: Evidence from savings rates, health behaviors, and retirement assets. American Economic Review, 103(2), 690–731. 29524925 10.1257/aer.103.2.690

[cogs13224-bib-0015] Chen, S. , Cronqvist, H. , Ni, S. , & Zhang, F. (2017). Languages and corporate savings behavior. Journal of Corporate Finance, 46, 320–341.

[cogs13224-bib-0016] Chi, J. D. , Su, X. , Tang, Y. , & Xu, B. (2018). Is language an economic institution? Evidence from R&D investment [online]. SSRN Electronic Journal, 1–48. 10.2139/ssrn.3262822

[cogs13224-bib-0017] Collins, M. (2002). Discriminative training methods for hidden Markov models: Theory and experiments with perceptron algorithms. In *Proceedings of the conference on empirical methods in natural language processing (EMNLP)* (pp. 1–8)., Philadelphia, PA. Association for Computational Linguistics.

[cogs13224-bib-0018] Condoravdi, C. (2002). Temporal interpretation of modals: Modals for the present and for the past. In D. Beaver , S. Kaufmann , B. Clark , & L. Casillas (Eds.), The construction of meaning (pp. 59–88). Stanford, CA: CSLI Publications

[cogs13224-bib-0019] Dahl, Ö. (1985). Tense and aspect systems. Oxford, England: Blackwell.

[cogs13224-bib-0020] Dahl, Ö. (Ed.). (2000a) . Tense and aspect in the languages of Europe. Berlin, Germany: Mouton de Gruyter.

[cogs13224-bib-0021] Dahl, Ö . (2000b). The grammar of future time reference in European languages. In Ö. Dahl (Ed. ), Tense and aspect in the languages of Europe (pp. 309–328). Berlin, Germany: Mouton de Gruyter

[cogs13224-bib-0022] Dahl, Ö. (2013). Stuck in the futureless zone. *Diversity Linguistics Comment*. https://dlc.hypotheses.org/360

[cogs13224-bib-0023] Du, W. , Green, L. , & Myerson, J. (2002). Cross‐cultural comparisons of discounting delayed and probabilistic rewards. The Psychological Record, 52, 479–492.

[cogs13224-bib-0024] Enç, M. (1996). Tense and modality. In S. Lappin (Ed. ), The handbook of contemporary semantic theory (pp. 345–58). Oxford, England: Blackwell

[cogs13224-bib-0025] Evans, N. , Bergqvist, H. , & San Roque, L. (2018). The grammar of engagement I: Framework and initial exemplification. Language and Cognition, 10(1), 110–140.

[cogs13224-bib-0026] Everett, C. (2013). Linguistic relativity: Evidence across languages and cognitive domains. Berlin, Germany: de Gruyter.

[cogs13224-bib-0027] Explosion AI (2020). spaCy. https://spacy.io/

[cogs13224-bib-0028] Fasan, M. , Gotti, G. , Kang, T. , & Liu, Y. (2016). Language FTR and earnings management: International evidence [Online]. SSRN Electronic Journal, 1–49. 10.2139/ssrn.2763922

[cogs13224-bib-0029] Fehringer, C. (2018). Internal constraints on the use of *gaan* versus *zullen* as future markers in spoken Dutch. Nederlandse Taalkunde, 22(3), 359–387.

[cogs13224-bib-0030] Figlio, D. , Giuliano, P. , Özek, U. , & Sapienza, P. (2016). Long‐term orientation and educational performance (Working Paper Series, 10147). Cambridge, MA: National Bureau of Economic Research.

[cogs13224-bib-0031] Fries, C. C. (1956). The expression of the future. Language Learning, 7(3‐4), 125–133.

[cogs13224-bib-0032] Galor, O. , Özak, Ö. , & Sarid, A. (2016). Geographical origins and economic consequences of language structures (IZA Discussion Paper Series, 10379). Bonn, Germany: Institute of Labor Economics.

[cogs13224-bib-0033] Garami, J. , & Moustafa, A. A. (2019). Probability discounting of monetary gains and losses in opioid‐dependent adults. Behavioural Brain Research, 364, 334–339. 30768996 10.1016/j.bbr.2019.02.017

[cogs13224-bib-0034] Geerts, G. , Haeseryn, W. , Romijn, K. , de Rooij, J. , & van den Toorn, M. (Eds.). (1997). 18.5.4.3.iii Gaan (2nd ed.). Retrieved from https://e‐ans.ivdnt.org/topics/pid/ans1805040303lingtopic

[cogs13224-bib-0035] Giannakidou, A. (2014). The futurity of the present and the modality of the future: A commentary on Broekhuis and Verkuyl. Natural Language and Linguistic Theory, 32, 1011–1032.

[cogs13224-bib-0036] Giannakidou, A. (2017). Epistemic future and epistemic MUST: Nonveridicality, evidence, and partial knowledge. In J. Blaszczak , A. Giannakidou , D. Klimek‐Jankowska , & K. Migdalski (Eds.), Mood, aspect, modality revisited: New answers to old questions. Chicago, IL: University of Chicago Press. 10.7208/chicago/9780226363660.003.0003

[cogs13224-bib-0037] Giannakidou, A. , & Mari, A. (2018). A unified analysis of the future as epistemic modality: The view from Greek and Italian. Natural Language and Linguistic Theory, 36(1), 85–129.

[cogs13224-bib-0038] Gotti, G. , Roberts, S. G. , Fasan, M. , & Robertson, C. (2021). Language in economics and accounting research: The role of linguistic history. The International Journal of Accounting, 56(03), 2150015.

[cogs13224-bib-0039] Green, L. , & Myerson, J. (2004). A discounting framework for choice with delayed and probabilistic rewards. Psychological Bulletin, 130(5), 769–792. 15367080 10.1037/0033-2909.130.5.769 PMC1382186

[cogs13224-bib-0040] Green, L. , Myerson, J. , & Ostaszewski, P. (1999). Amount of reward has opposite effects on the discounting of delayed and probabilistic outcomes. Journal of Experimental Psychology: Learning, Memory, and Cognition, 25(2), 418–427. 10093208 10.1037//0278-7393.25.2.418

[cogs13224-bib-0041] Green, L. , Myerson, J. , & Vanderveldt, A. (2014). Delay and probability discounting. In *The Wiley Blackwell handbook of operant and classical conditioning* (Chap. 13, pp. 374–409). Hoboken, NJ: Wiley.

[cogs13224-bib-0042] Guin, B. (2017). Culture and household saving (Working Paper Series, 2069, pp. 1–53). Frankfurt, Germany: European Central Bank.

[cogs13224-bib-0043] Gumperz, J. J. , & Levinson, S. C. (Eds.). (1996). Rethinking linguistic relativity. Cambridge, England: Cambridge University Press.

[cogs13224-bib-0044] Hamilton, K. R. , & Potenza, M. N. (2012). Relations among delay discounting, addictions, and money mismanagement: Implications and future directions. American Journal of Drug and Alcohol Abuse, 38(1), 30–42. 22211535 10.3109/00952990.2011.643978 PMC3691101

[cogs13224-bib-0045] Hodgins, D. C. , & Engel, A. (2002). Future time perspective in pathological gamblers. Journal of Nervous and Mental Disease, 190(11), 775–780. 12436018 10.1097/00005053-200211000-00008

[cogs13224-bib-0046] Hopper, P. J. (1996). Some recent trends in grammaticalization. Annual Review of Anthropology, 25(1), 217–236.

[cogs13224-bib-0047] Hü bner, M. , & Vannoorenberghe, G. (2015a). Patience and inflation (No. 65811). Munich Personal RePEc Archive.

[cogs13224-bib-0048] Hübner, M. , & Vannoorenberghe, G. (2015b). Patience and long‐run growth. Economics Letters, 137, 163–167.

[cogs13224-bib-0049] Huddleston, R. (1995). The case against a future tense in English. Studies in Language, 19(2), 399–446.

[cogs13224-bib-0050] Huddleston, R. , & Pullum, G. K. (2002). The Cambridge grammar of the English language. Cambridge, England: Cambridge University Press.

[cogs13224-bib-0051] Ireland, M. E. , Schwartz, H. A. , Chen, Q. , Ungar, L. H. , & Albarracín, D. (2015). Future‐oriented tweets predict lower county‐level HIV prevalence in the United States. Health Psychology, 34(Supplement), 1252–1260. 10.1037/hea0000279 PMC5621637 26651466

[cogs13224-bib-0052] Jakobson, R. (1971). Selected writings, volume II. The Hague, The Netherlands: Mouton.

[cogs13224-bib-0053] Janssen, T. (1989). Die Hilfsverben *werden* (Deutsch) und *zullen* (Niederländisch): Modal oder temporal? In W. Abraham & T. Janssen (Eds.), *Tempus‐Aspekt‐Modus: Die lexikalischen und grammatischen Formen in den germanischen Sprachen* (pp. 65–84). Tübingen, The Netherlands: Neimeyer.

[cogs13224-bib-0054] Karawani, H. , & Waldon, B. (2017). May or might? Strength, duality and social meaning. In Cremers, A. , van Gessel, T. , & Roelofsen, F. (Eds.), Proceedings of the 21st Amsterdam colloquium (pp. 305–314)., Amsterdam, The Netherlands: Institute for Logic, Language, and Computation.

[cogs13224-bib-0055] Kim, B. K. , & Zauberman, G. (2009). Perception of anticipatory time in temporal discounting. Journal of Neuroscience, Psychology, and Economics, 2(2), 91–101.

[cogs13224-bib-0056] Kim, J. , Kim, Y. , & Zhou, J. (2017). Languages and earnings management. Journal of Accounting and Economics, 63(2‐3), 288–306.

[cogs13224-bib-0057] Kirby, K. N. , Petry, N. M. , & Bickel, W. K. (1999). Heroin addicts have higher discount rates for delayed rewards than non‐drug‐using controls. Journal of Experimental Psychology: General, 128(1), 78–87. 10100392 10.1037//0096-3445.128.1.78

[cogs13224-bib-0058] Kirsner, R. S. (1969). The role of *zullen* in the grammar of modern standard Dutch. Lingua, 24, 101–154.

[cogs13224-bib-0059] Klecha, P. (2014). Diagnosing modality in predictive expressions. Journal of Semantics, 31(3), 443–455.

[cogs13224-bib-0060] Klein, W. (1995). A time‐relational analysis of Russian aspect. Language, 71(4), 669–695.

[cogs13224-bib-0061] Koo, T. , Carreras, X. , & Collins, M. (2008). Simple semi‐supervised dependency parsing. In *Proceedings of ACL‐08: HLT*, (pp. 595–603). Columbus, OH: Association for Computational Linguistics.

[cogs13224-bib-0062] Kratzer, A. (1977). What ‘must’ and ‘can’ must and can mean. Linguistics and Philosophy, 1(3), 337–355.

[cogs13224-bib-0063] Kratzer, A. (2012). Modality and conditionals: New and revised perspectives. Oxford, England: Oxford University Press.

[cogs13224-bib-0064] Lassiter, D. (2015). Epistemic comparison, models of uncertainty, and the disjunction puzzle. Journal of Semantics, 32(4), 649–684.

[cogs13224-bib-0065] Leavitt, J. (2011). Linguistic relativities. Cambridge, England: Cambridge University Press.

[cogs13224-bib-0066] Legler, J. , & Roback, P. (2019). Generalized linear models and multilevel models. Boston, MA: Bookdown.

[cogs13224-bib-0067] Liang, H. , Marquis, C. , Renneboog, L. , & Sun, S. L. (2018). Future‐time framing: The effect of language on corporate future orientation. Organization Science, 29(6), 1093–1111.

[cogs13224-bib-0068] Lien, D. , & Zhang, S. (2020). Words matter life: The effect of language on suicide behavior. Journal of Behavioral and Experimental Economics, 86, 1–12.

[cogs13224-bib-0069] Liu, W. , & Aaker, J. (2007). Do you look to the future or focus on today? The impact of life experience on intertemporal decisions. Organizational Behavior and Human Decision Processes, 102(2), 212–225.

[cogs13224-bib-0070] Luckman, A. , Donkin, C. , & Newell, B. R. (2018). Can a single model account for both risky choices and inter‐temporal choices? Testing the assumptions underlying models of risky inter‐temporal choice. Psychonomic Bulletin and Review, 25(2), 785–792. 28600719 10.3758/s13423-017-1330-8

[cogs13224-bib-0071] Lucy, J. A. (1992). Language diversity and thought. Cambridge, England: Cambridge University Press.

[cogs13224-bib-0072] Lucy, J. A. (1997). Linguistic relativity. Annual Review of Anthropology, 26, 291–312.

[cogs13224-bib-0073] Lucy, J. A. (2016). Recent advances in the study of linguistic relativity in historical context: A critical assessment. Language Learning, 66(3), 487–515.

[cogs13224-bib-0074] Lüdecke, D. (2019). ggeffects: Marginal effects of regression models. https://strengejacke.github.io/ggeffects/

[cogs13224-bib-0075] Lupyan, G. , Rahman, R. A. , Boroditsky, L. , & Clark, A. (2020). Effects of language on visual perception. PsyArxiv. 10.1016/j.tics.2020.08.005 33012687

[cogs13224-bib-0076] Lyons, J. (1968). Grammatical categories. In Introduction to theoretical linguistics (pp. 270–333). Cambridge, England: Cambridge University Press.

[cogs13224-bib-0077] Maas, C. J. , & Hox, J. J. (2004). Robustness issues in multilevel regression analysis. Statistica Neerlandica, 58(2), 127–137.

[cogs13224-bib-0078] Majid, A. (2018). Language and cognition. In H. Callan (Ed. ), The international encyclopedia of anthropology. Hoboken, NJ: Wiley, Ltd.

[cogs13224-bib-0079] Mavisakalyan, A. , Tarverdi, Y. , & Weber, C. (2018). Talking in the present, caring for the future: Language and environment. Journal of Comparative Economics, 46(4), 1370–1387.

[cogs13224-bib-0080] Mavisakalyan, A. , & Weber, C. (2018). Linguistic structures and economic outcomes. Journal of Economic Surveys, 32(3), 916–939.

[cogs13224-bib-0081] McKerchar, T. L. , & Renda, C. R. (2012). Delay and probability discounting in humans: An overview. Psychological Record, 62(4), 817–834.

[cogs13224-bib-0082] McWhorter, J. H. (2014). The language hoax: Why the world looks the same in any language. Oxford, England: Oxford University Press.

[cogs13224-bib-0083] Mejía‐Cruz, D. , Green, L. , Myerson, J. , Morales‐Chainé, S. , & Nieto, J. (2016). Delay and probability discounting by drug‐dependent cocaine and marijuana users. Psychopharmacology, 233(14), 2705–2714. 27180181 10.1007/s00213-016-4316-8

[cogs13224-bib-0084] Mezhevich, I. (2008). A Time‐relational approach to tense and mood. In Proceedings of the West Coast conference on formal linguistics (Vol. 27, pp. 326–334)., Somerville, MA. Cascadilla Proceedings Project.

[cogs13224-bib-0085] Nuyts, J. (2000). Epistemic modality, language, and conceptualization. Amsterdam, The Netherlands: John Benjamins.

[cogs13224-bib-0086] Nuyts, J. , & Vonk, W. (1999). Epistemic modality and focus in Dutch. Linguistics, 37(4), 699.

[cogs13224-bib-0087] Olmen, D. V. , Mortelmans, T. , & Auwera, J. V. D. (2009). Grammaticalization and subjectification of the future: The case of English, Dutch and German. In W. Olesky & P. Stalmaszcyk (Eds.), Cognitive approaches to language and linguistic data: Studies in honor of Barbara Lewandowska‐Tomaszczyk (pp. 285–306). Frankfurt, Germany: Peter Lang AG

[cogs13224-bib-0088] Palmer, R. F. (2001). Mood and modality (2 ed.). Cambridge, England: Cambridge University Press.

[cogs13224-bib-0089] Pereltsvaig, A. (2011). You save what you speak? *Languages of the World*. https://www.languagesoftheworld.info/language-and-mind/you-save-what-you-speak.html

[cogs13224-bib-0090] Pérez, E. O. , & Tavits, M. (2017). Language shapes people's time perspective and support for future‐oriented policies. American Journal of Political Science, 61(3), 715–727.

[cogs13224-bib-0091] Pullum, G. K. (2012). Keith Chen, Whorfian economist. Language Log. http://languagelog.ldc.upenn.edu/nll/?p=3756

[cogs13224-bib-0092] Python Software Foundation (2017). Python language reference. http://www.python.org

[cogs13224-bib-0093] Rachlin, H. , Raineri, A. , & Cross, D. (1991). Subjective probability and delay. Journal of the Experimental Analysis of Behavior, 55(2), 233–44. 2037827 10.1901/jeab.1991.55-233 PMC1323057

[cogs13224-bib-0094] Raudenbush, S. , & Bryk, A. (2002). Hierarchical linear models: Applications and data analysis and methods. London, England: Sage.

[cogs13224-bib-0095] Roberts, S. , Winters, J. , & Chen, K. (2015). Future tense and economic decisions: Controlling for cultural evolution. PLoS ONE, 10(7), 1–46. 10.1371/journal.pone.0132145 PMC4506144 26186527

[cogs13224-bib-0096] Royster, J. F. , & Steadman, J. M. J. (1923). The “going‐to” future. In J. M. Manly (Ed. ), The Manly anniversary studies in language and literature (pp. 394—‐403). Chicago, IL: University of Chicago Press

[cogs13224-bib-0097] Salkie, R. (2010). *Will*: Tense or modal or both? English Language and Linguistics, 14(2), 187–215.

[cogs13224-bib-0098] Sarkar, A. (1998). The conflict between future tense and modality: The case of *will* in English. University of Pennsylvania Working Papers in Linguistics, 5(2), 91–117.

[cogs13224-bib-0099] Sedivy, J. (2012). Thought experiments on language and thought. Language Log. https://languagelog.ldc.upenn.edu/nll/?p=3797

[cogs13224-bib-0100] Sluijs, R. V. (2011). The modal system of 20th century Negerhollands (Unpublished doctoral dissertation). Radboud University, Nijmegen, The Netherlands.

[cogs13224-bib-0101] Sutter, M. , Angerer, S. , Glätzle‐rützler, D. , & Lergetporer, P. (2015). The effect of language on economic behavior: Experimental evidence from children's intertemporal choices (IZA Discussion Paper Series no. 9383, pp. 1–47). Bonn, Germany: Institute for Labor Economics.

[cogs13224-bib-0102] Tate, L. M. , Tsai, P. F. , Landes, R. D. , Rettiganti, M. , & Lefler, L. L. (2015). Temporal discounting rates and their relation to exercise behavior in older adults. Physiology and Behavior, 152(Pt. A), 295–299. 26440317 10.1016/j.physbeh.2015.10.003

[cogs13224-bib-0103] Te Winkel, L. A. (1866). Over de wijzen en tijden der werkwoorden. De Taalgids, 8(66‐75).

[cogs13224-bib-0104] Ten Cate, A. P. (1991). Bemerkungen zum deutschen und niederländischen Futur. In Klein, E. (Ed.), Betriebslinguistik und Linguistikbetrieb, Linguistisches Kolloquium, volume 24, (pp. 23‐31)., Tübingen, The Netherlands. Niemeyer.

[cogs13224-bib-0105] Thoma, D. , & Tytus, A. E. (2018). How cross‐linguistic differences in the grammaticalization of future time reference influence intertemporal choices. Cognitive Science, 42(3), 974–1000. 10.1111/cogs.12525 28833400

[cogs13224-bib-0106] Thorstad, R. , Nie, A. , & Wolff, P. (2015). Representations of time affect willingness to wait for future rewards. In Proceedings of the annual meeting of the Cognitive Science Society (pp. 2392–2397). Austin, TX: Cognitive Science Society.

[cogs13224-bib-0107] Thorstad, R. , & Wolff, P. (2018). A big data analysis of the relationship between future thinking and decision‐making. Proceedings of the National Academy of Sciences, 115, E1741–E1748. 10.1073/pnas.1706589115 PMC5828570 29432182

[cogs13224-bib-0108] Twisk, J. (2006). Applied multilevel analysis: A practical guide. Cambridge, England: Cambridge University Press.

[cogs13224-bib-0109] Vanderveldt, A. , Green, L. , & Myerson, J. (2015). Discounting of monetary rewards that are both delayed and probabilistic: Delay and probability combine multiplicatively, not additively. Journal of Experimental Psychology: Learning Memory and Cognition, 41(1), 148–162. 24933696 10.1037/xlm0000029 PMC4268098

[cogs13224-bib-0110] Vanderveldt, A. , Green, L. , & Rachlin, H. (2017). Discounting by probabilistic waiting. Journal of Behavioral Decision Making, 30(1), 39–53.

[cogs13224-bib-0111] Verkuyl, H. J. (2008). Binary tense. In *Vol. 187 of CSLI lecture notes*. Stanford, VA: CSLI Publications.

[cogs13224-bib-0112] Vuchinich, R. E. , & Simpson, C. A. (1998). Hyperbolic temporal discounting in social drinkers and problem drinkers. Experimental and Clinical Psychopharmacology, 6(3), 292–305. 9725113 10.1037//1064-1297.6.3.292

[cogs13224-bib-0113] Whorf, B. (1956). Language, thought, and reality: Selected writings of Benjamin Lee Whorf. Cambridge, MA: MIT Press.

[cogs13224-bib-0114] Wolff, P. , & Holmes, K. J. (2011). Linguistic relativity. Wiley Interdisciplinary Reviews: Cognitive Science, 2(3), 253–265. 26302074 10.1002/wcs.104

[cogs13224-bib-0115] World Values Survey Association (2014). World values survey: Round three (v. 20180912). Vienna, Austria: Author.

[cogs13224-bib-0116] Xiao, J. J. , & Porto, N. (2019). Present bias and financial behavior. Financial Planning Review, 2(2), 1–14.

[cogs13224-bib-0117] Zauberman, G. , Kim, B. K. , Malkoc, S. A. , & Bettman, J. R. (2009). Discounting time and time discounting: Subjective time perception and intertemporal preferences. Journal of Marketing Research, 46(4), 543–556.

